# Supplementary material for: Genetic predisposition to hypertension is associated with preeclampsia in European and Central Asian women
Source: Nat Commun. 2020 Nov 25;11:5976. doi: 10.1038/s41467-020-19733-6 (PMC7688949; doi:10.1038/s41467-020-19733-6)
Supplement: Supplementary file 1 — Supplementary Information [file 41467_2020_19733_MOESM1_ESM.pdf]

# Supplementary Information

## Genetic predisposition to hypertension is associated with preeclampsia in European and Central Asian women

Steinthorsdottir, McGinnis, Williams and Stefansdottir et al.

## Contents


| | Title | Page |
|---|---|---|
| Supplementary Figures | | |
| Supplementary Figure 1 | Manhattan plots of population specific meta-analyses | 3 |
| Supplementary Figure 2 | Power to detect genome-wide significant association using the current meta-analyses | 4 |
| Supplementary Figure 3 | Forest plots of variants associating with preeclampsia through fetal or maternal genome | 5 |
| Supplementary Figure 4 | Locus plots for variants associating with preeclampsia through fetal or maternal genome | 8 |
| Supplementary Figure 5 | Comparison of effect estimates for blood pressure risk variants in preeclampsia and blood pressure traits | 10 |
| Supplementary Figure 6 | Ancestry Principal Components of Central Asia compared to European and Asian 1000 Genomes Populations | 11 |
| Supplementary Figure 7 | Imputation performance for Chromosome 1 vs Central Asia MAF | 12 |
| Supplementary Figure 8 | Distribution of genome coverage by mapped reads for Kazakh samples and Uzbek samples | 13 |
| Supplementary Figure 9 | Proportion of recovered 1000 Genomes SNPs versus Central Asia GATK Variant Calling Threshold (VQSLOD) | 14 |
| Supplementary Figure 10 | Efficacy of Genotype Refinement | 15 |
| Supplementary Figure 11 | Per Sample Concordance vs Per Sample Coverage | 16 |
| Supplementary Figure 12 | Imputation performance for Chromosome 1 vs Europe MAF | 17 |
| Supplementary Figure 13 | Imputation performance for Chromosome 1 vs East Asia MAF | 18 |
| Supplementary Figure 14 | Imputation performance for Chromosome 1 vs South Asia MAF | 19 |

| Supplementary Tables | | |
|---|---|---|
| Supplementary Table 1 | Studies included in meta-analyses, follow-up and downstream analyses | 20 |
| Supplementary Table 2 | Follow up of variants with *P* < 1E-6 in the offspring meta-analysis | 22 |
| Supplementary Table 3 | Conditional analysis chr13 locus | 22 |
| Supplementary Table 4 | Correlation (r2) between variants at the chr13 *FLT1* locus reported in this study and previous report [PMID: 28628106] | 23 |
| Supplementary Table 5 | Correlation in European and Kazakh samples between variants with the lowest *P*-value in the maternal preeclampsia meta-analysis and blood pressure variants reported in Evangelou et al. 2018 [PMID: 30224653] | 23 |
| Supplementary Table 6 | PE or GH risk allele concordance with high BP allele at 892 other known BP variants | 23 |
| Supplementary Table 7 | Results for associated variants in offspring and maternal and discovery meta-analyses | 24 |
| Supplementary Table 8 | Samples included in preeclampsia subgroup analysis | 24 |
| Supplementary Table 9 | Heritability of preeclampsia | 25 |
| Supplementary Table 10 | Effect of preeclampsia variants on gestational hypertension | 25 |
| Supplementary Table 11 | Genetic correlation between maternal preeclampsia and selected secondary traits | 26 |
| Supplementary Table 12 | Polygenic risk score analysis | 27 |
| Supplementary Table 13 | Clinical characteristics | 28 |
| Supplementary Table 14 | Secondary traits from deCODE and UKBB | 30 |

| Supplementary Notes: | | |
|---|---|---|
| Supplementary Note 1 | Construction of Central Asia Haplotype Reference Panel | 33 |
| Supplementary Note 2 | Correcting for Population Stratification in EMIM | 37 |
| Supplementary Note 3 | Study Acknowledgements and Funding | 41 |

| Supplementary References | | 42 |
|---|---|---|

a.

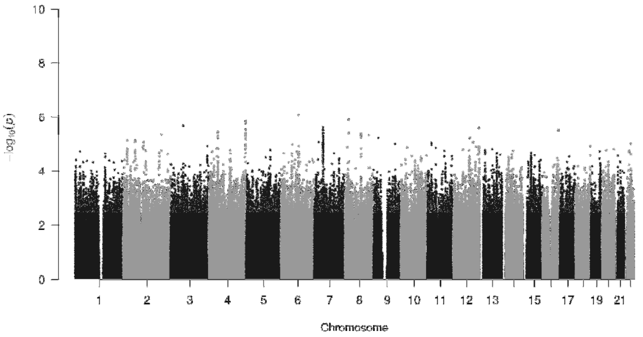


b.

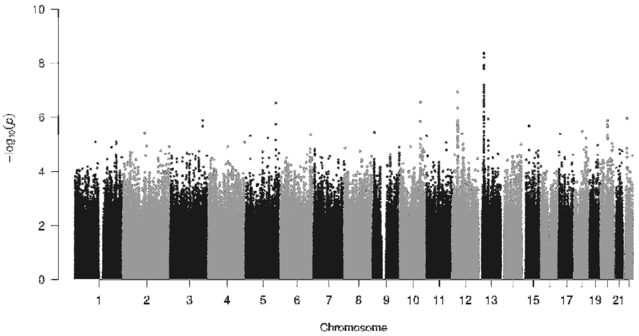


c.

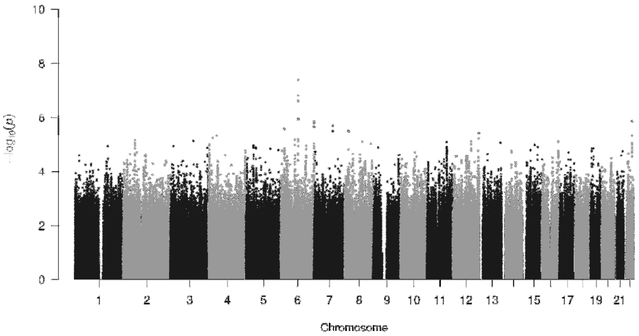


d.

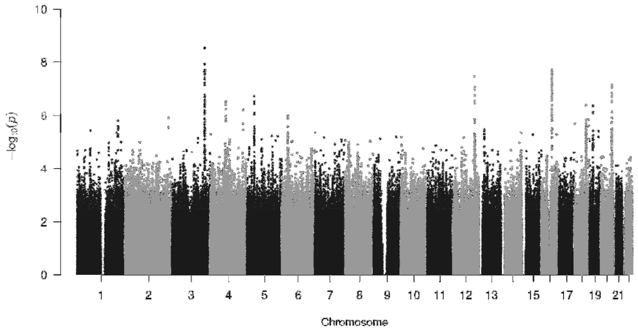


**Supplementary Figure 1 Manhattan plots of population specific meta-analyses**

Panel a. Central Asian preeclampsia offspring, b. European preeclampsia offspring, c. Central Asian preeclampsia mothers, d. European preeclampsia mothers.

a.

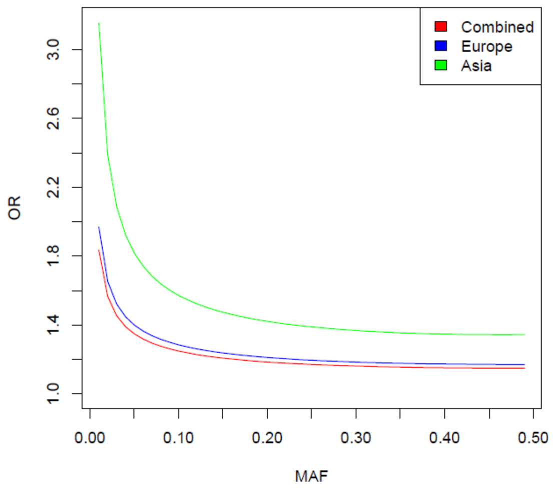


b.

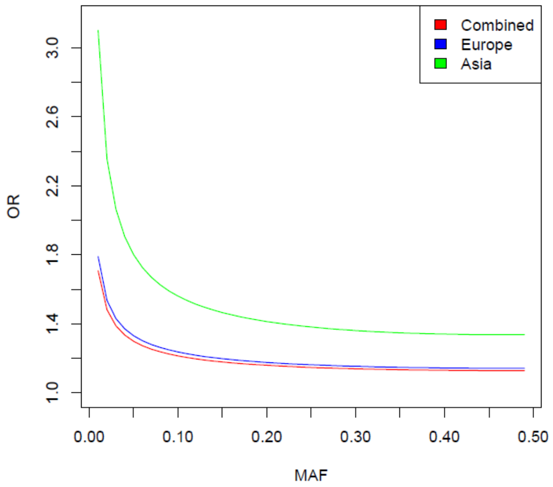


**Supplementary Figure 2 Power to detect genome-wide significant association using the current meta-analyses**

The plots show allelic odds ratio (OR) required for 80% power to detect association to preeclampsia at genome-wide significance ($P < 4 \times 10^{-9}$ after adjusting for 12 million variants tested) in meta-analysis of offspring or maternal subjects from Europe, Central Asia or Europe and Central Asia combined. Results are shown for a causal SNP with minor allele frequency (MAF) in the range of 0.01-0.5.

a. Offspring meta-analyses. Effective sample size for the Central-Asian, European and Combined meta-analysis is 2,064, 7,259 and 9,323 cases respectively and an equal number of controls. In the combined analysis we have 80% power to detect an OR > 1.15 at MAF = 0.5.
b. Maternal meta-analyses. Effective sample size for the Central-Asian, European and Combined meta-analysis is 2,137, 10,255 and 12,392 cases respectively and an equal number of controls. In the combined analysis we have 80% power to detect an OR > 1.13 at MAF = 0.5.

**a.**

**rs4769612[C] *FLT1*/13q12**

| Study | Cases | Controls | OR | 95% CI | P-value |
|---|---|---|---|---|---|
| **GWAS** | | | | | |
| deCODE | 2,221 | 360,005 | 1.17 | (1.07-1.29) | $1.2\times10^{-3}$ |
| GOPEC | 1,004 | 5,286 | 1.23 | (1.11-1.35) | $3.2\times10^{-5}$ |
| ALSPAC | 143 | 6,039 | 1.14 | (0.90-1.44) | 0.28 |
| MoBa | 1,046 | 961 | 1.12 | (0.99-1.27) | 0.080 |
| SSI | 213 | 963 | 1.28 | (1.03-1.59) | 0.028 |
| Kazakhstan I | 789 | 795 | 1.08 | (0.94-1.25) | 0.27 |
| Kazakhstan II | 542 | 349 | 1.18 | (0.97-1.43) | 0.10 |
| Uzbekistan | 814 | 883 | 1.21 | (1.06-1.38) | $5.8\times10^{-3}$ |
| **META** | **6,772** | **375,281** | **1.17** | **(1.12-1.23)** | **$3.0\times10^{-11}$** |
| **Follow-up** | | | | | |
| FINNPEC * | 580 | 760 | 1.27 | (1.09-1.48) | $2.5\times10^{-3}$ |
| Kazakhstan | 452 | 361 | 1.27 | (1.03-1.56) | 0.028 |
| **META** | **1,032** | **1,121** | **1.27** | **(1.12-1.44)** | **$1.9\times10^{-4}$** |
| **Combined** | **7,804** | **376,402** | **1.19** | **(1.13-1.24)** | **$4.3\times10^{-14}$** |

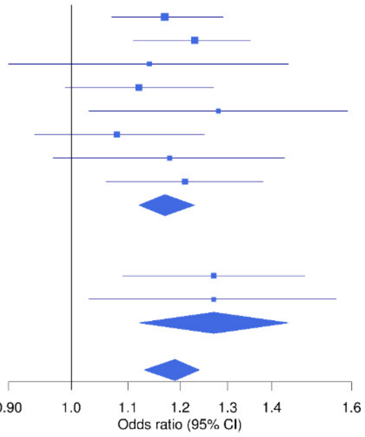


**rs1421085[C] *FTO*/16q12**

| Study | Cases | Controls | OR | 95% CI | P-value |
|---|---|---|---|---|---|
| **GWAS** | | | | | |
| deCODE | 2,453 | 134,837 | 1.15 | (1.07-1.23) | $9.5\times10^{-5}$ |
| GOPEC | 1,875 | 5,088 | 1.04 | (0.96-1.13) | 0.30 |
| ALSPAC | 150 | 6,058 | 0.94 | (0.74-1.18) | 0.58 |
| MoBa | 1,469 | 954 | 1.19 | (1.06-1.33) | $4.1\times10^{-3}$ |
| SSI | 872 | 815 | 1.23 | (1.08-1.41) | $2.2\times10^{-3}$ |
| FINNRISK | 400 | 7,805 | 1.25 | (1.08-1.46) | $3.8\times10^{-3}$ |
| Kazakhstan I | 846 | 814 | 1.20 | (1.03-1.39) | 0.018 |
| Kazakhstan II | 640 | 361 | 1.17 | (0.96-1.42) | 0.12 |
| Uzbekistan | 810 | 884 | 1.01 | (0.87-1.17) | 0.92 |
| **META** | **9,515** | **157,616** | **1.13** | **(1.08-1.17)** | **$2.0\times10^{-9}$** |
| **Follow-up** | | | | | |
| FINNPEC * | 678 | 700 | 1.16 | (0.99-1.36) | 0.065 |
| HUNT | 1,030 | 4,264 | 1.06 | (0.96-1.17) | 0.26 |
| DNBC | 335 | 1,054 | 1.01 | (0.84-1.22) | 0.89 |
| Kazakhstan | 592 | 361 | 1.00 | (0.80-1.23) | 0.96 |
| **META** | **2,635** | **6,379** | **1.06** | **(0.99-1.14)** | **0.089** |
| **Combined** | **12,150** | **163,995** | **1.11** | **(1.07-1.15)** | **$1.2\times10^{-9}$** |

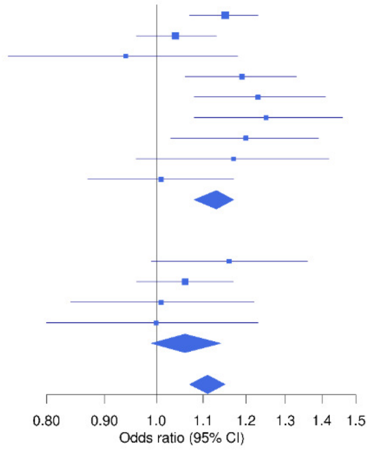


**rs259983[C] *ZNF831*/20q13**

| Study | Cases | Controls | OR | 95% CI | P-value |
|---|---|---|---|---|---|
| **GWAS** | | | | | |
| deCODE | 2,453 | 134,837 | 1.20 | (1.10-1.32) | $9.9\times10^{-5}$ |
| GOPEC | 1,875 | 5,088 | 1.16 | (1.04-1.30) | $7.0\times10^{-3}$ |
| ALSPAC | 150 | 6,058 | 1.05 | (0.75-1.47) | 0.80 |
| MoBa | 1,469 | 954 | 1.15 | (0.99-1.35) | 0.073 |
| SSI | 872 | 815 | 1.07 | (0.89-1.28) | 0.47 |
| FINNRISK | 400 | 7,805 | 1.20 | (0.99-1.44) | 0.057 |
| Kazakhstan I | 846 | 814 | 1.16 | (0.90-1.49) | 0.26 |
| Kazakhstan II | 640 | 361 | 0.89 | (0.62-1.26) | 0.50 |
| Uzbekistan | 810 | 884 | 1.07 | (0.85-1.36) | 0.56 |
| **META** | **9,515** | **157,616** | **1.15** | **(1.09-1.22)** | **$2.2\times10^{-7}$** |
| **Follow-up** | | | | | |
| HUNT | 1,030 | 4,264 | 1.26 | (1.10-1.45) | $8.8\times10^{-4}$ |
| DNBC * | 335 | 1,054 | 1.14 | (0.89-1.47) | 0.30 |
| Kazakhstan | 592 | 361 | 1.39 | (0.96-1.99) | 0.078 |
| **META** | **1,957** | **5,679** | **1.25** | **(1.11-1.40)** | **$1.4\times10^{-4}$** |
| **Combined** | **11,472** | **163,295** | **1.17** | **(1.11-1.23)** | **$2.9\times10^{-10}$** |

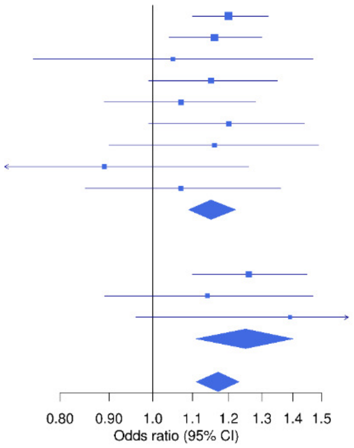

**b.**

### rs1918975[T] *MECOM*/3q26

| Study | Cases | Controls | OR | 95% CI | P-value |
|---|---|---|---|---|---|
| **GWAS** | | | | | |
| deCODE | 2,453 | 134,837 | 1.13 | (1.05-1.21) | $7.7 \times 10^{-4}$ |
| GOPEC | 1,875 | 5,088 | 1.14 | (1.05-1.23) | $1.1 \times 10^{-3}$ |
| ALSPAC | 150 | 6,058 | 1.00 | (0.79-1.27) | 1.00 |
| MoBa | 1,469 | 954 | 1.12 | (1.00-1.26) | 0.048 |
| SSI | 872 | 815 | 1.17 | (1.02-1.35) | 0.026 |
| FINNRISK | 400 | 7,805 | 1.12 | (0.97-1.29) | 0.13 |
| Kazakhstan I | 846 | 814 | 1.03 | (0.89-1.19) | 0.68 |
| Kazakhstan II | 640 | 361 | 1.12 | (0.93-1.36) | 0.23 |
| Uzbekistan | 810 | 884 | 1.07 | (0.93-1.23) | 0.33 |
| **META** | **9,515** | **157,616** | **1.12** | **(1.07-1.16)** | $\mathbf{2.5 \times 10^{-8}}$ |
| **Follow-up** | | | | | |
| FINNPEC | 678 | 700 | 0.98 | (0.84-1.15) | 0.83 |
| HUNT | 1,030 | 4,264 | 1.13 | (1.02-1.25) | 0.019 |
| DNBC | 335 | 1,054 | 1.03 | (0.86-1.23) | 0.78 |
| Kazakhstan | 592 | 361 | 1.00 | (0.83-1.22) | 0.97 |
| **META** | **2,635** | **6,379** | **1.06** | **(0.99-1.14)** | **0.094** |
| **Combined** | **12,150** | **163,995** | **1.10** | **(1.07-1.14)** | $\mathbf{1.2 \times 10^{-8}}$ |

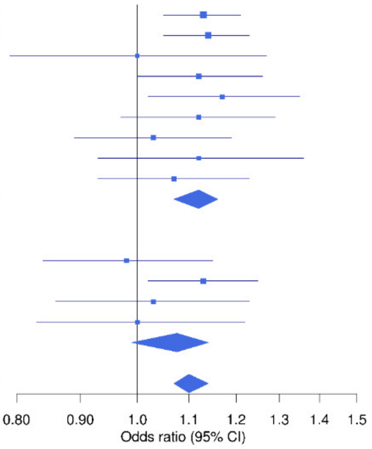


### rs1458038[T] *FGF5*/4q21

| Study | Cases | Controls | OR | 95% CI | P-value |
|---|---|---|---|---|---|
| **GWAS** | | | | | |
| deCODE | 2,453 | 134,837 | 1.09 | (1.02-1.18) | 0.014 |
| GOPEC | 1,875 | 5,088 | 1.11 | (1.03-1.21) | 0.010 |
| ALSPAC | 150 | 6,058 | 1.44 | (1.13-1.83) | $3.0 \times 10^{-3}$ |
| MoBa | 1,469 | 954 | 1.15 | (1.01-1.29) | 0.028 |
| SSI | 872 | 815 | 1.11 | (0.97-1.28) | 0.13 |
| FINNRISK | 400 | 7,805 | 1.14 | (0.98-1.33) | 0.10 |
| Kazakhstan I | 846 | 814 | 1.14 | (0.99-1.32) | 0.063 |
| Kazakhstan II | 640 | 361 | 1.21 | (1.00-1.45) | 0.045 |
| Uzbekistan | 810 | 884 | 1.00 | (0.87-1.15) | 1.00 |
| **META** | **9,515** | **157,616** | **1.12** | **(1.07-1.16)** | $\mathbf{4.2 \times 10^{-8}}$ |
| **Follow-up** | | | | | |
| FINNPEC | 678 | 700 | 1.15 | (0.98-1.35) | 0.080 |
| HUNT | 1,030 | 4,264 | 1.05 | (0.95-1.17) | 0.31 |
| DNBC | 335 | 1,054 | 1.01 | (0.83-1.21) | 0.94 |
| Kazakhstan | 592 | 361 | 1.10 | (0.91-1.34) | 0.33 |
| **META** | **2,635** | **6,379** | **1.07** | **(1.00-1.15)** | **0.063** |
| **Combined** | **12,150** | **163,995** | **1.11** | **(1.07-1.15)** | $\mathbf{1.2 \times 10^{-8}}$ |

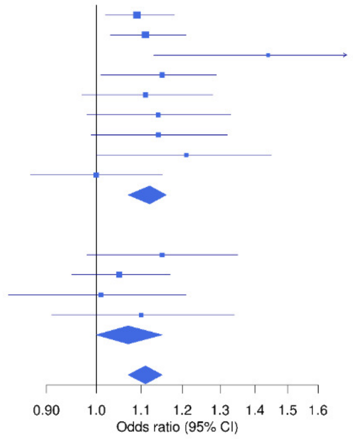


### rs10774624[G] *SH2B3*/12q24

| Study | Cases | Controls | OR | 95% CI | P-value |
|---|---|---|---|---|---|
| **GWAS** | | | | | |
| deCODE | 2,453 | 134,837 | 1.12 | (1.05-1.20) | $1.1 \times 10^{-3}$ |
| GOPEC | 1,875 | 5,088 | 1.06 | (0.98-1.14) | 0.16 |
| ALSPAC | 150 | 6,058 | 1.21 | (0.96-1.52) | 0.11 |
| MoBa | 1,469 | 954 | 1.19 | (1.06-1.34) | $4.2 \times 10^{-3}$ |
| SSI | 872 | 815 | 1.26 | (1.09-1.46) | $1.6 \times 10^{-3}$ |
| FINNRISK | 400 | 7,805 | 1.16 | (1.00-1.35) | 0.055 |
| Kazakhstan I | 846 | 814 | 1.05 | (0.87-1.26) | 0.61 |
| Kazakhstan II | 640 | 361 | 1.06 | (0.83-1.35) | 0.65 |
| Uzbekistan | 810 | 884 | 1.04 | (0.89-1.21) | 0.63 |
| **META** | **9,515** | **157,616** | **1.12** | **(1.07-1.16)** | $\mathbf{7.0 \times 10^{-8}}$ |
| **Follow-up** | | | | | |
| HUNT | 1,030 | 4,264 | 1.04 | (0.94-1.15) | 0.49 |
| DNBC * | 335 | 1,054 | 1.18 | (0.99-1.41) | 0.061 |
| Kazakhstan | 592 | 361 | 1.15 | (0.90-1.48) | 0.27 |
| **META** | **1,957** | **5,679** | **1.08** | **(0.99-1.17)** | **0.07** |
| **Combined** | **11,472** | **163,295** | **1.11** | **(1.07-1.15)** | $\mathbf{1.7 \times 10^{-8}}$ |

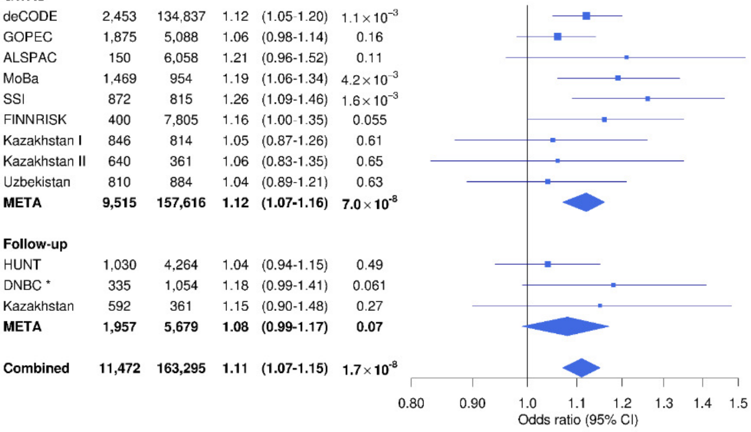

**Supplementary Figure 3 Forest plots of variants associating with preeclampsia through fetal or maternal genome.**

Variant name followed by risk allele and locus name are presented at the top. Name of study, number of cases and controls, odds ratio (OR) and *P*-value are indicated. For each variant results are presented for each GWAS dataset and each follow-up dataset. META indicates meta-analysis results for GWAS datasets, follow-up datasets and Combined indicates the combined results for the GWAS and follow-up datasets. The first plot is based on association in preeclampsia offspring while the other five are based on association in maternal preeclampsia. Stars indicate that a proxy marker was used, for details see footnote in Supplementary Table 2 (offspring variants) and footnotes in Supplementary Data 2 (maternal variants). a. Variants with genome-wide significant association, b. Variants at loci validated through association with BP. Individual cohort *P*-values are obtained from logistic regression of case status on genotype count adjusted for covariates (see Methods). META and Combined *P*-values are obtained from fixed-effect meta-analysis of effects and *P*-values, adjusted for genomic control. All *P*-values are two-sided.

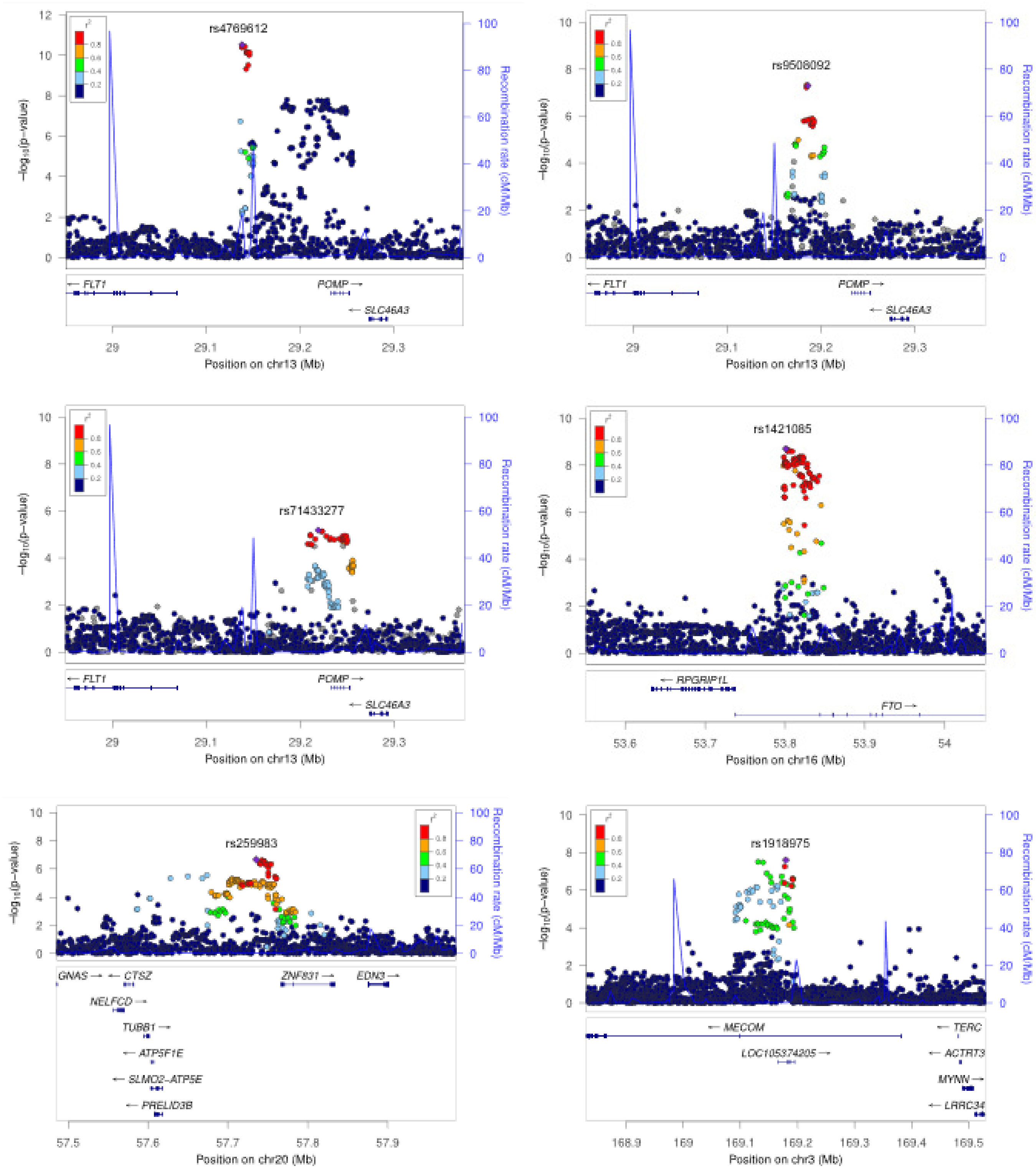
rs4769612
rs9508092
rs71433277
rs1421085
rs259983
rs1918975
−log10(p−value)
Recombination rate (cM/Mb)
FLT1
POMP
SLC46A3
RPGRIP1L
FTO
GNAS
CTSZ
NELFCD
TUBB1
ATP5F1E
SLMO2−ATP5E
PRELID3B
ZNF831
EDN3
MECOM
LOC105374205
TERC
ACTRT3
MYNN
LRRC34
Position on chr13 (Mb)
Position on chr13 (Mb)
Position on chr13 (Mb)
Position on chr16 (Mb)
Position on chr20 (Mb)
Position on chr3 (Mb)

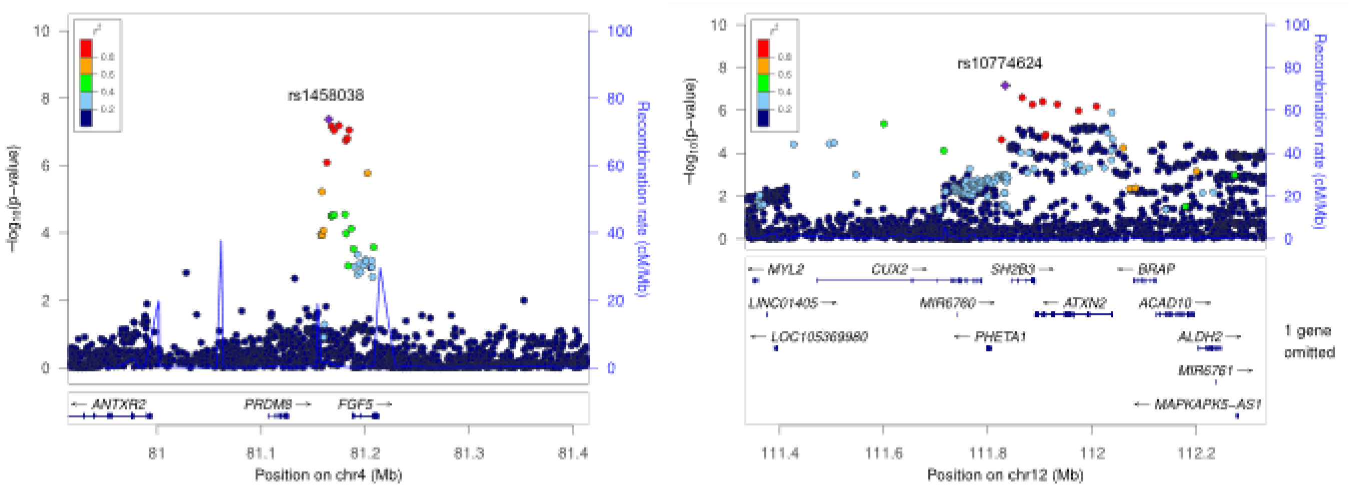


**Supplementary Figure 4 Locus plots for variants associating with preeclampsia through fetal or maternal genome.**

*P* values (-log10) of SNP associations in the meta-analysis are plotted against their chromosomal positions (NCBI Build 37 coordinates). Index variant is denoted by a purple diamond; other variants are colored to reflect their correlation with the index variant. Known genes in the region are shown underneath the plot. Secondary signals at the *FLT1*/13q12 locus: rs9508092 is shown after conditioning on rs4769612 and rs71433277 and rs71433277 is shown after conditioning on rs4769612 and rs9508092.

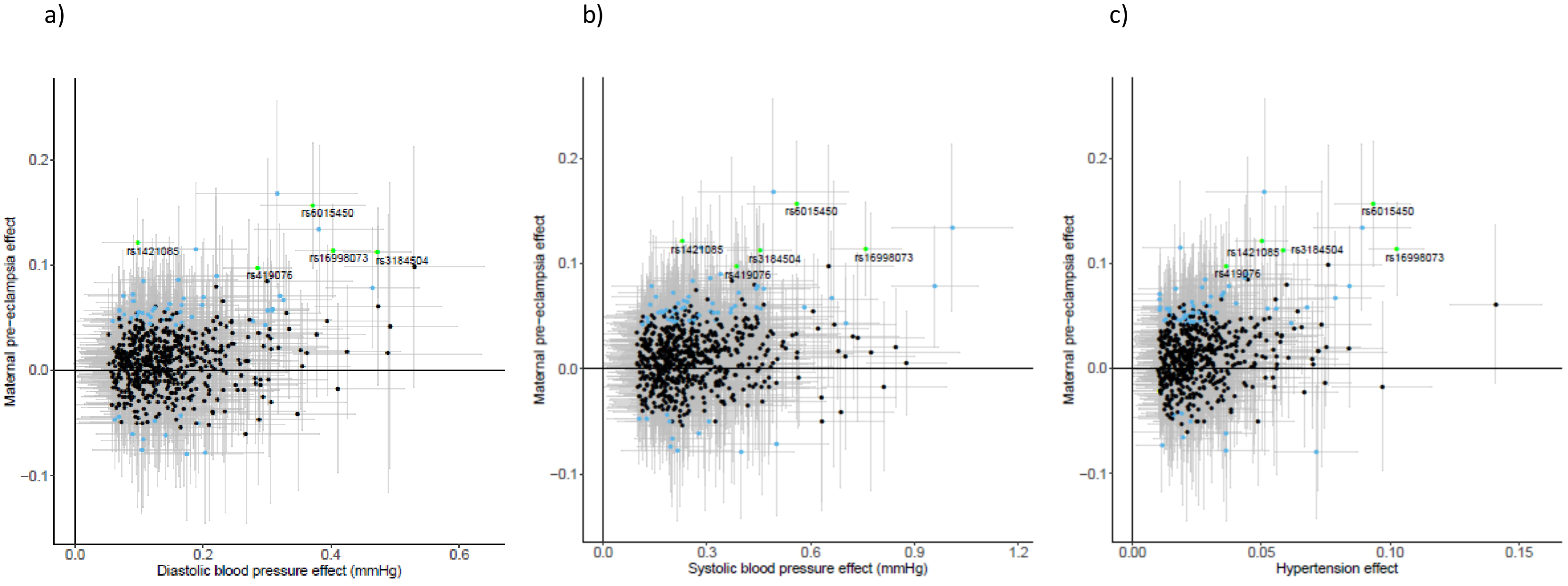


**Supplementary Figure 5 Comparison of effect estimates for BP risk variants in preeclampsia and BP traits**

The plots show variants reported to be associated with BP traits (Diastolic BP, Systolic BP or Pulse pressure) [Evangelou, E. *et al*. PMID: 30224653] and also highlight the five variants associated with preeclampsia in this study. Effect in meta-analysis of Icelandic and UK data sets of the allele associated with increased BP on a) Diastolic BP, b) Systolic BP and c) Hypertension (x-axis) vs. effect in European meta-analysis on maternal preeclampsia (y-axis). Crosses indicate 95% confidence intervals. Dots show only those variants with $P < 0.05$ for each BP trait and with MAF > 5% in our preeclampsia meta-analysis. Green dots and their corresponding rs ID denote the four variants reported here that associate with preeclampsia through BP. The fifth green dot denotes the *FTO* variant rs1421085 which is absent from the BP list used here which is based on BMI adjusted BP analysis. Blue dots denote other variants for which $P < 0.05$ for preeclampsia. Black dots denote variants with $P \geq 0.05$ for preeclampsia. The blood pressure and hypertension data are based on combined analyses of males and females in the Icelandic and UK datasets. Gender specific analyses gave similar point estimates for males and females.

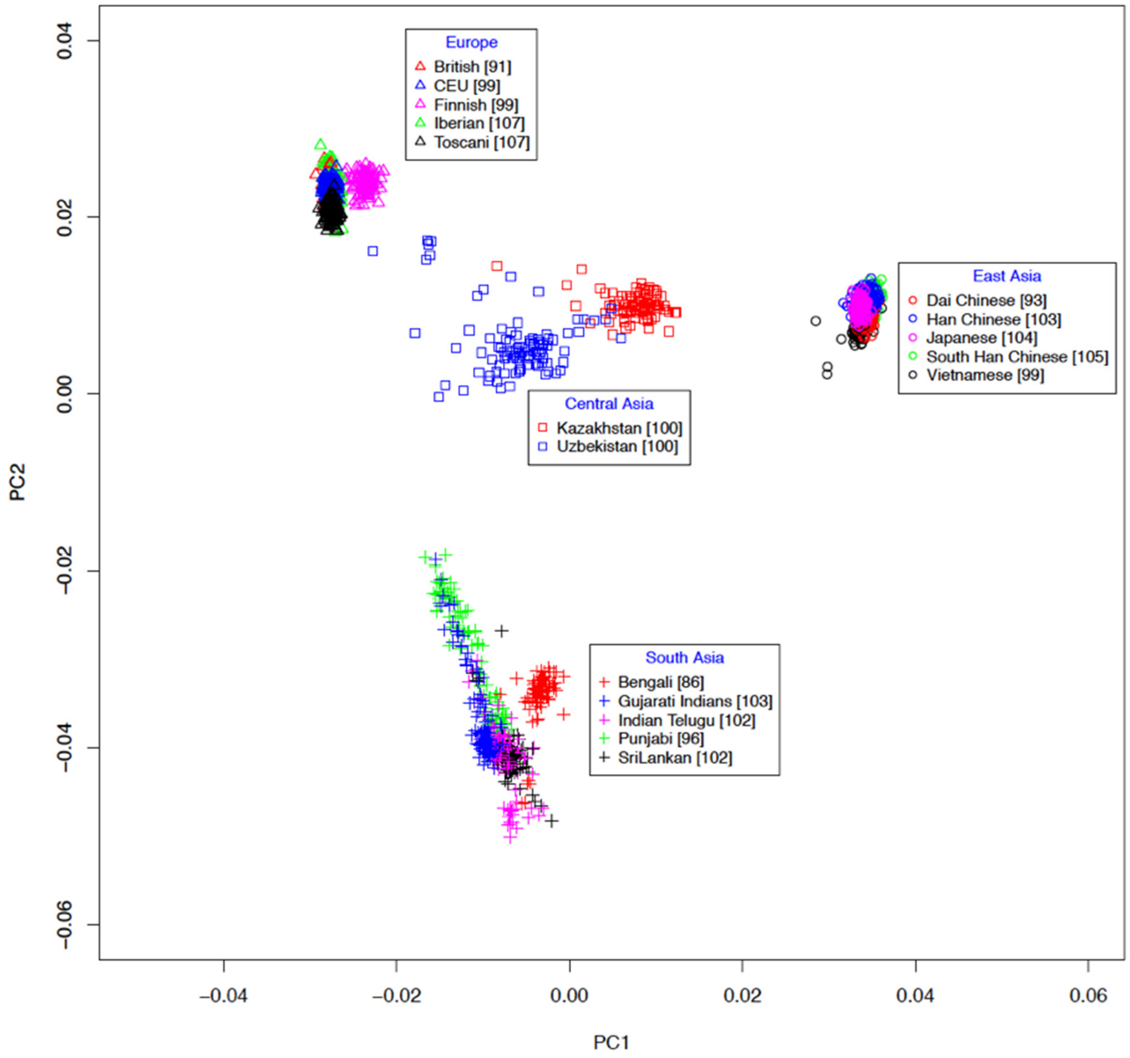


**Supplementary Figure 6. Ancestry Principal Components of Central Asia compared to European and Asian 1000 Genomes Populations**

The plot shows that the Kazakh and Uzbek populations in the context of European and Asian 1000 genomes populations. Both the Central Asian populations are on a cline between East Asia and Europe with Uzbekistan exhibiting a greater affinity with Europe and South Asia. The number of subjects in each ethnic/country grouping is shown in square brackets.

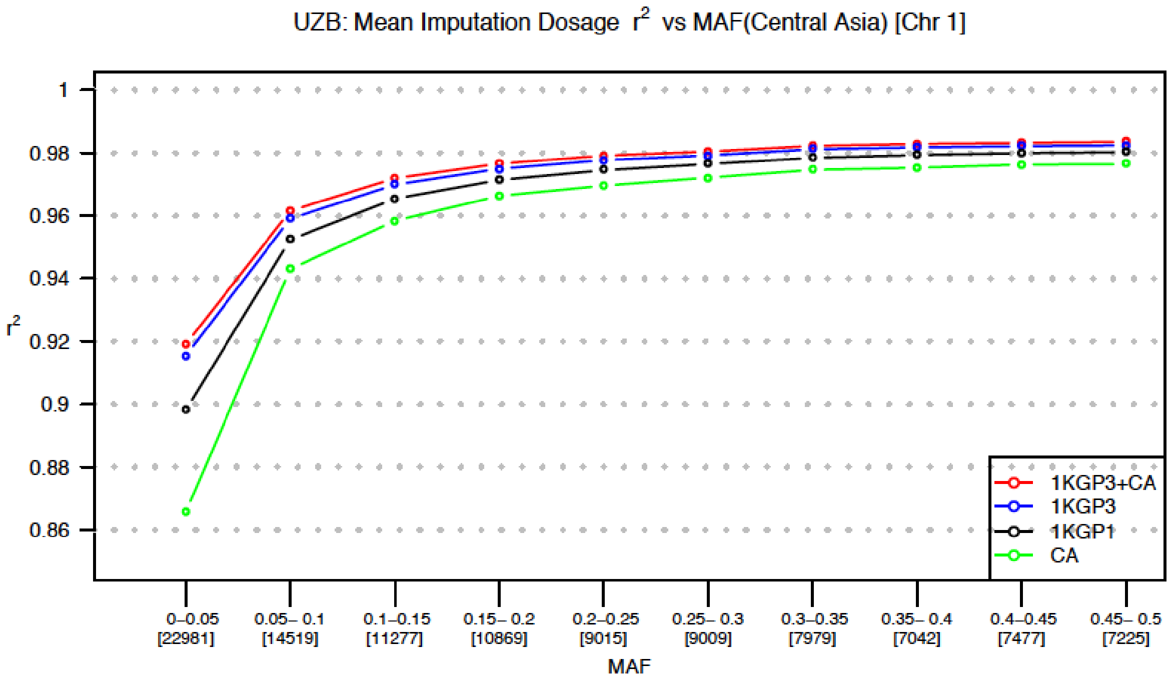


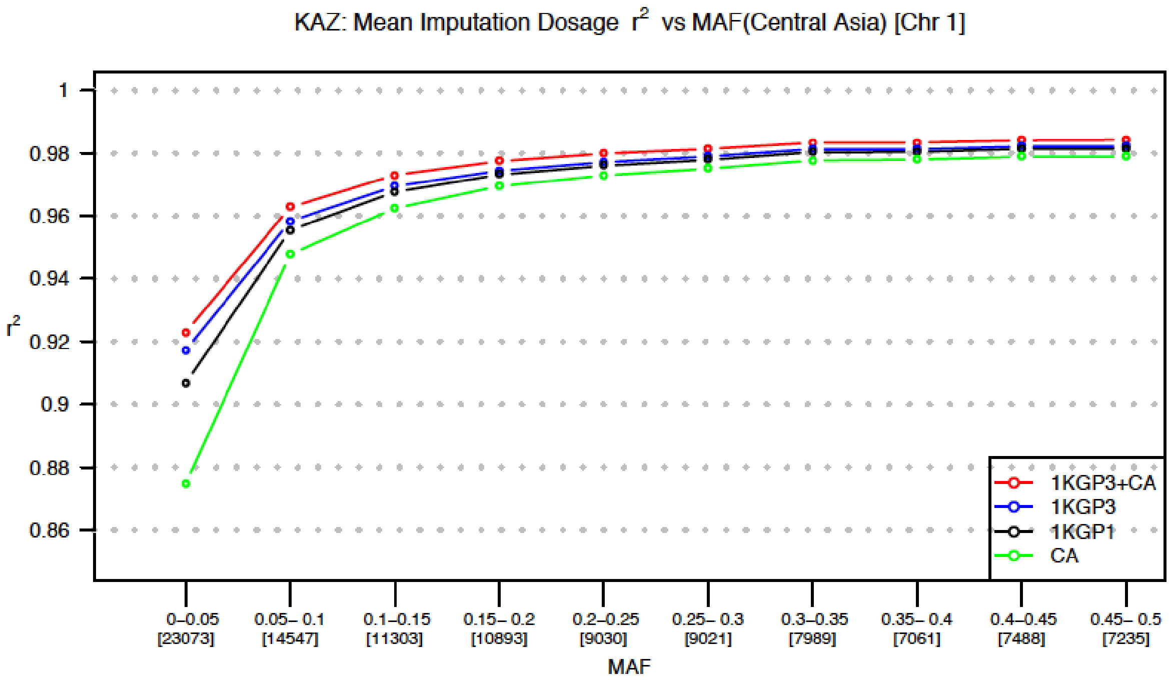


**Supplementary Figure 7: Imputation performance for Chromosome 1 vs Central Asia MAF**

The Minor Allele Frequency (MAF) is as calculated in the combined Central Asian WGS samples. The number of variants in each MAF bin is shown underneath the label in square brackets.

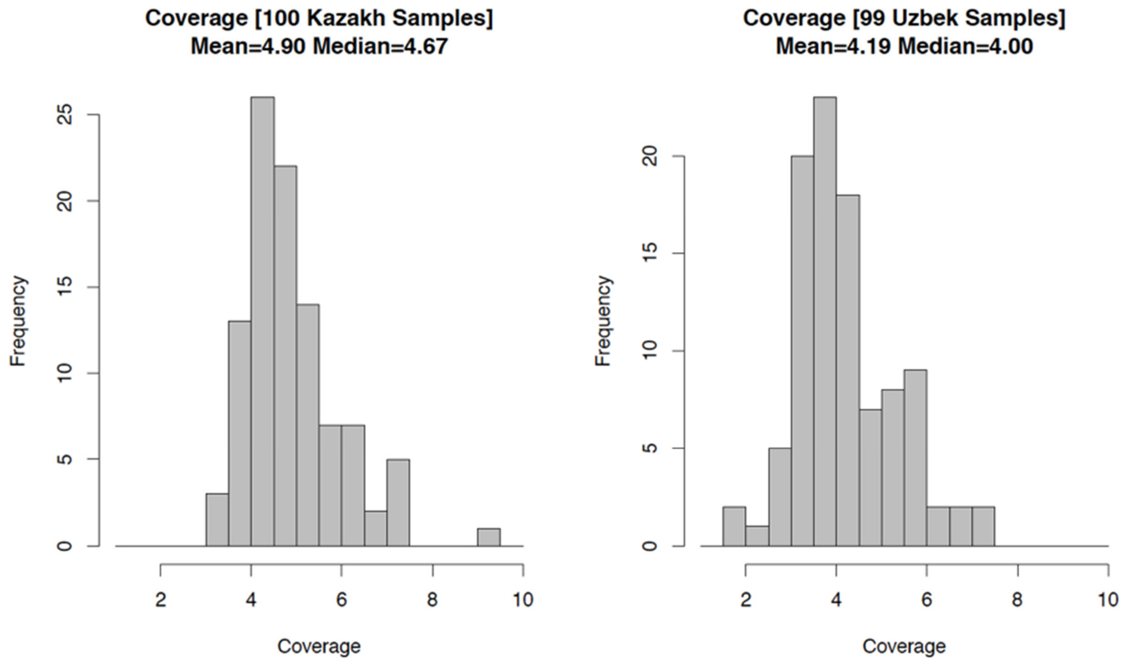


**Supplementary Figure 8: Distribution of genome coverage by mapped reads for Kazakh samples and Uzbek samples**

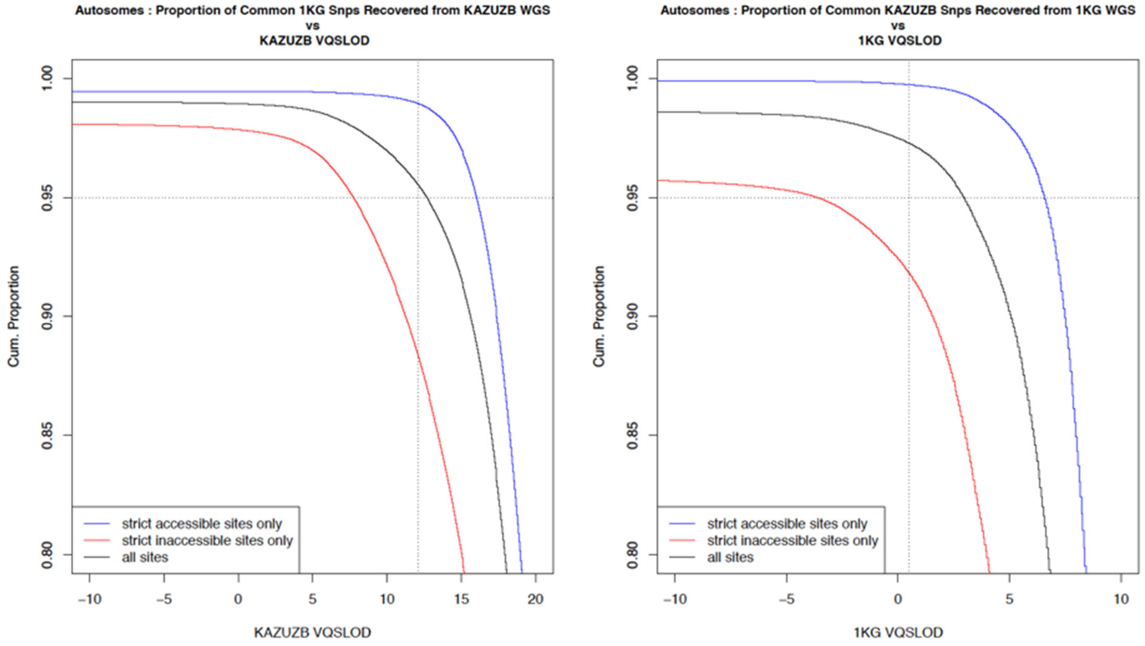


**Supplementary Figure 9: Proportion of recovered 1000 Genomes SNPs versus Central Asia GATK Variant Calling Threshold (VQSLOD)**

The plots show that variants that are common (maf > 10%) in all 1000 genomes regional populations and in the 1000 genomes accessible genomic regions are reliably discovered in our dataset. The 1000 Genomes VQSLOD was downloaded from ftp://ftp.1000genomes.ebi.ac.uk/vol1/ftp/phase1/analysis_results/consensus_call_sets/snps/ALL.wgs.LC_VQSR2b_EX_ILLUMINA_EX_SOLID_union.phase1.snps.lowcov_plus_exome.sites.vcf.gz. Accessibility masks were downloaded from ftp://ftp.1000genomes.ebi.ac.uk/vol1/ftp/phase1/analysis_results/supporting/accessible_genome_masks.

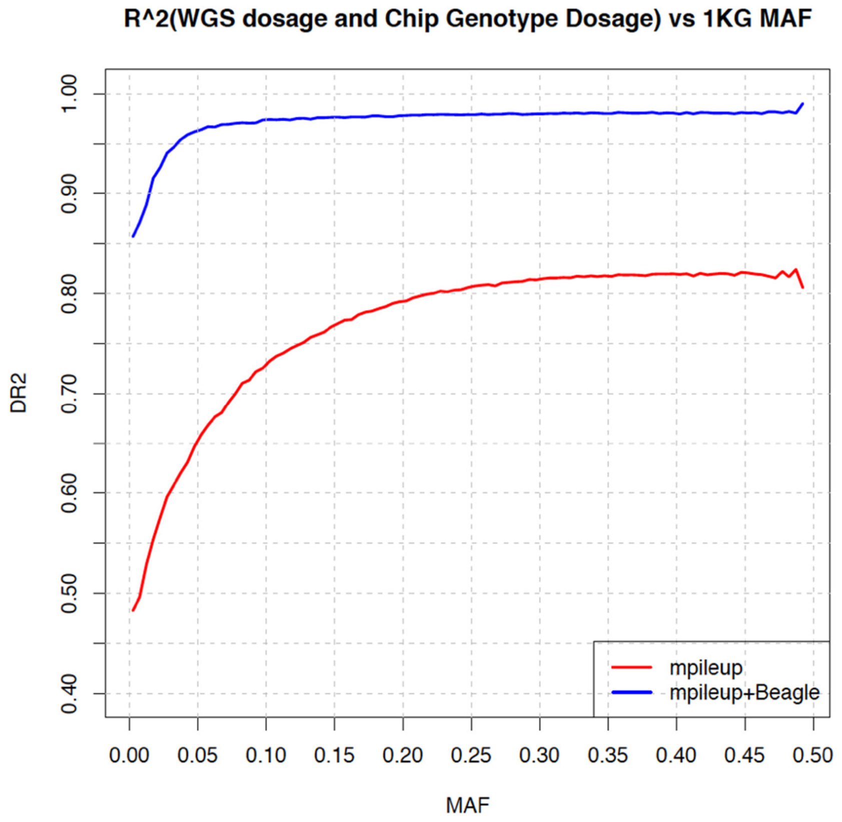


**Supplementary Figure 10. Efficacy of Genotype Refinement**

The plot shows the efficacy of the genotype refinement using Beagle and the 1000 genomes reference panel.

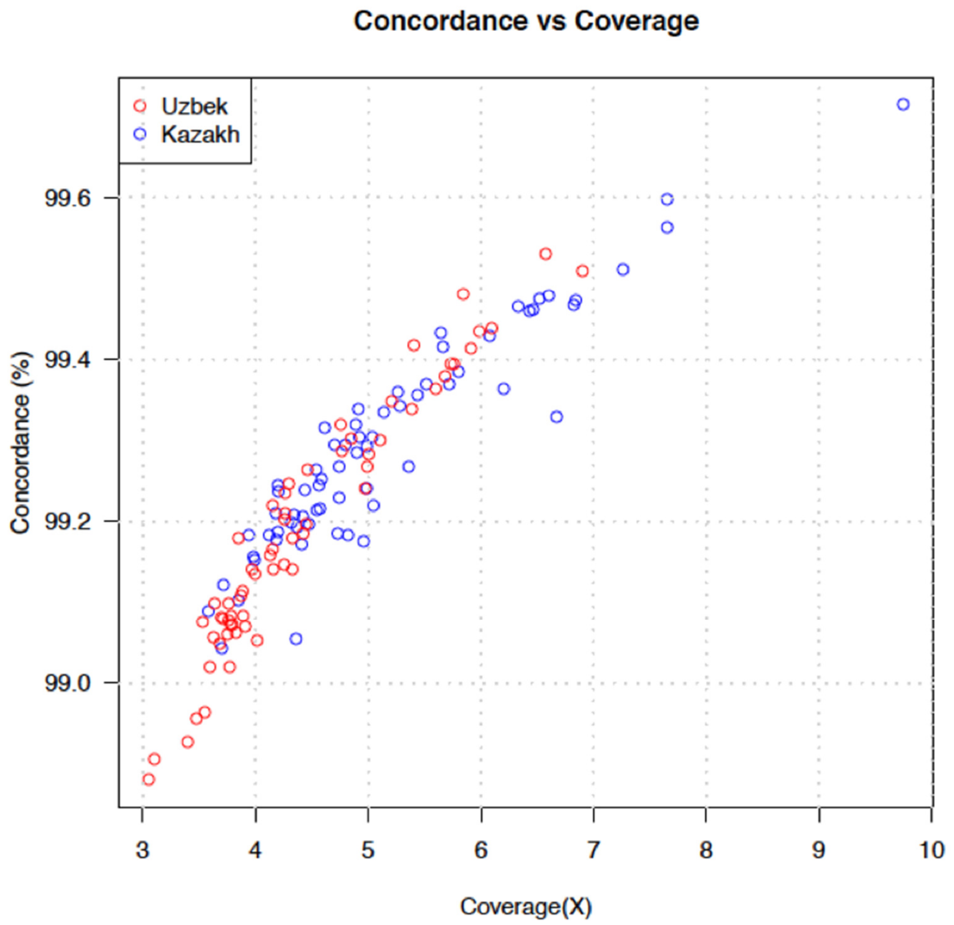


**Supplementary Figure 11: Per Sample Concordance vs Per Sample Coverage**

The plot shows that the concordance improves markedly with sample coverage. The concordance with chip genotypes is calculated using best guess genotypes with a genotype probability threshold of 0.6.

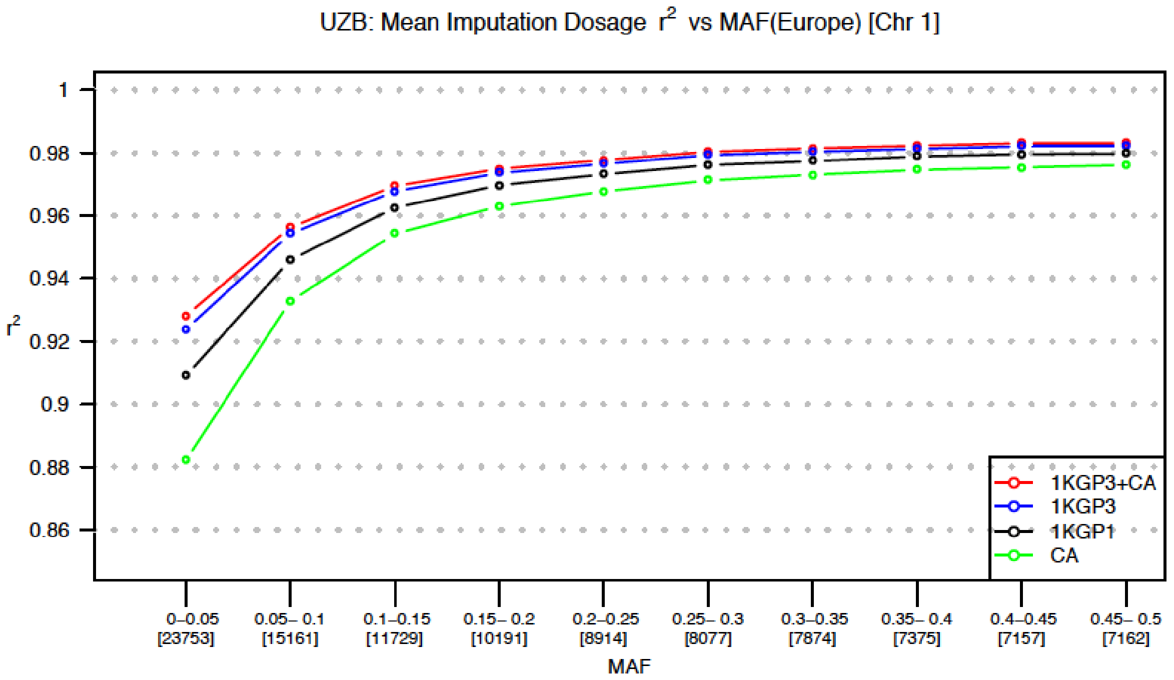


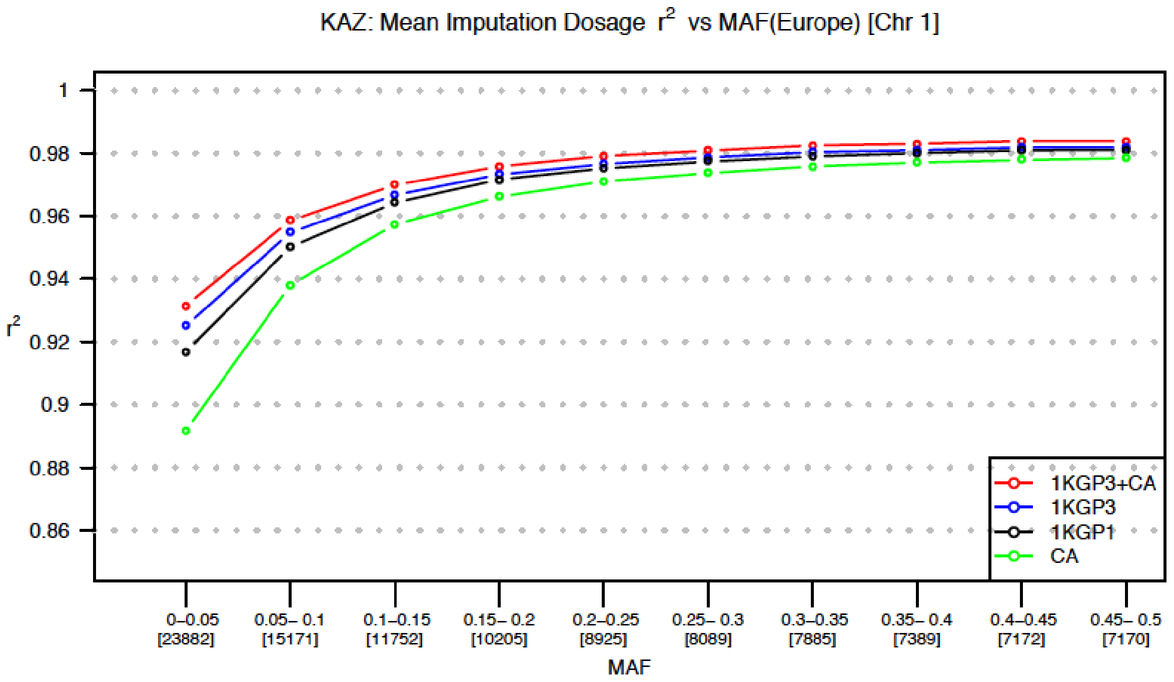


**Supplementary Figure 12: Imputation performance for Chromosome 1 vs Europe MAF**

The Minor Allele Frequency (MAF) is as calculated in the 1000 Genomes Phase 3 European samples. The number of variants in each MAF bin is shown underneath the label in square brackets.

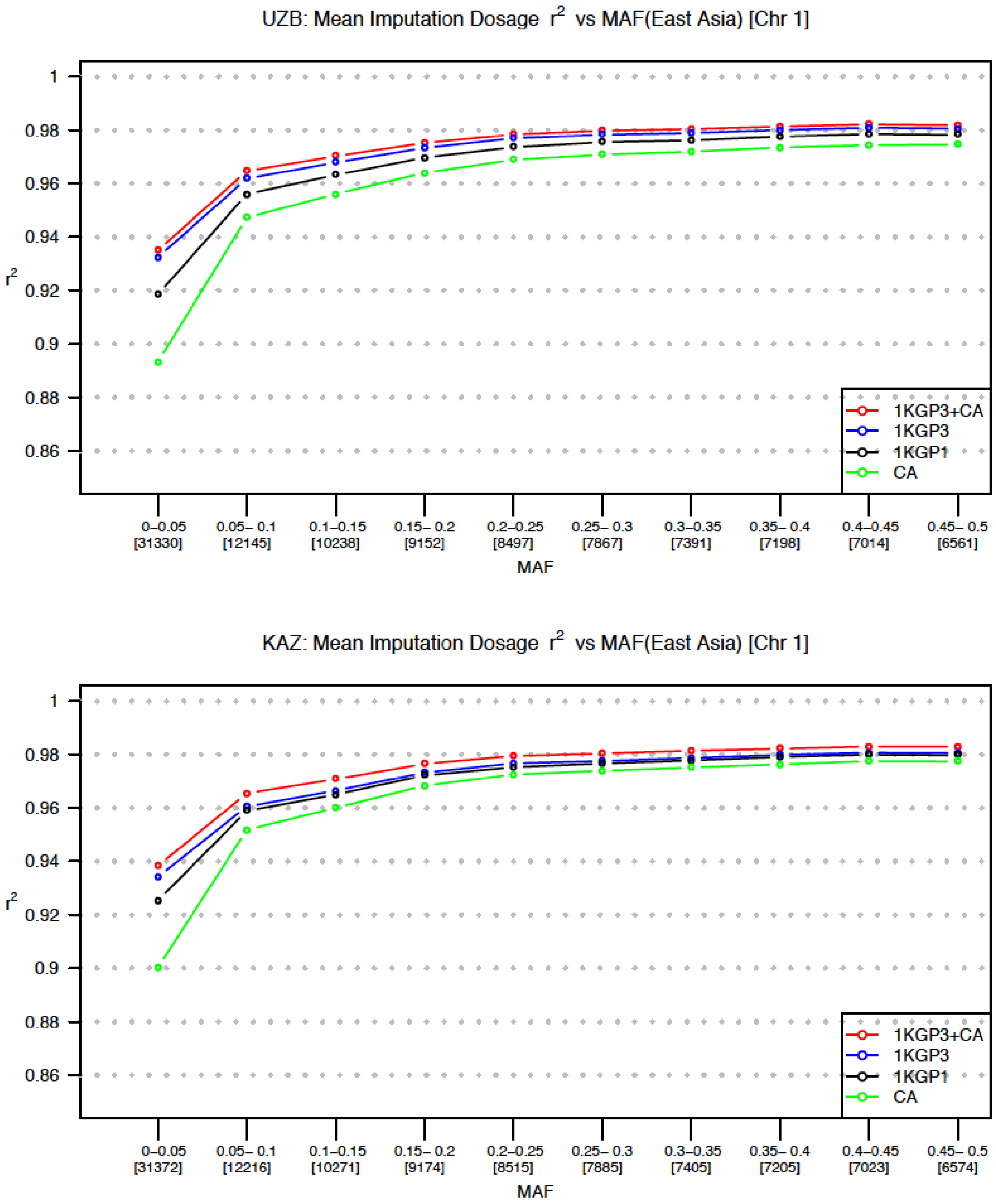


**Supplementary Figure 13: Imputation performance for Chromosome 1 vs East Asia MAF**

The Minor Allele Frequency (MAF) is as calculated in the 1000 Genomes Phase 3 East Asian samples. The number of variants in each MAF bin is shown underneath the label in square brackets.

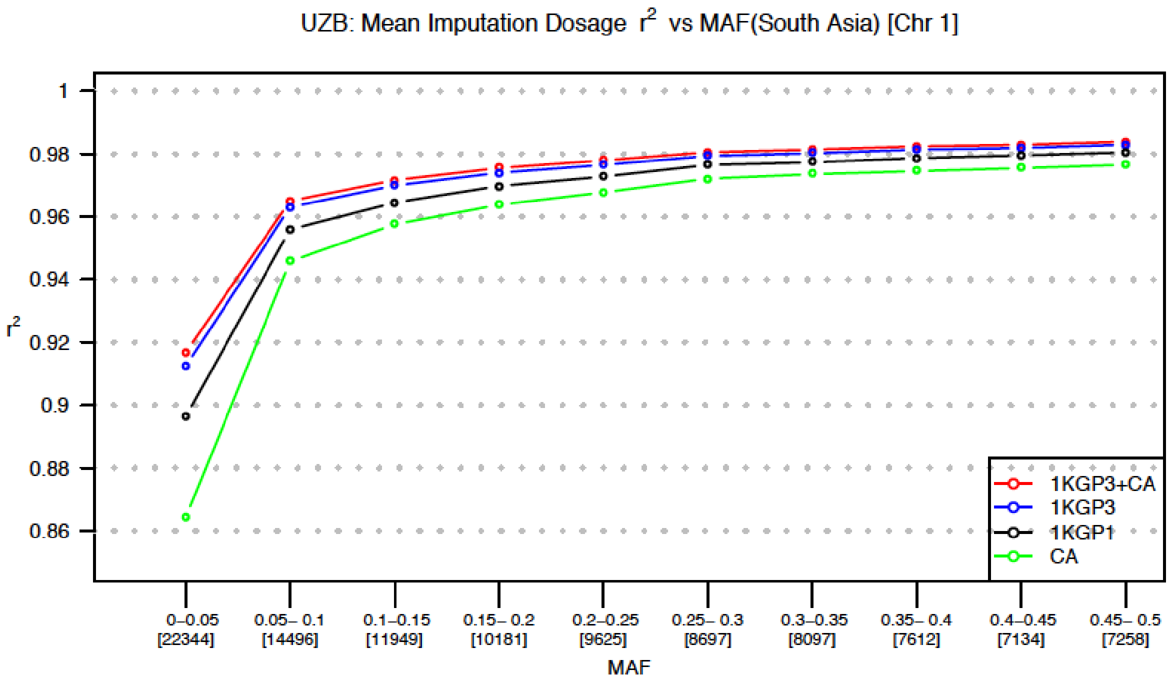


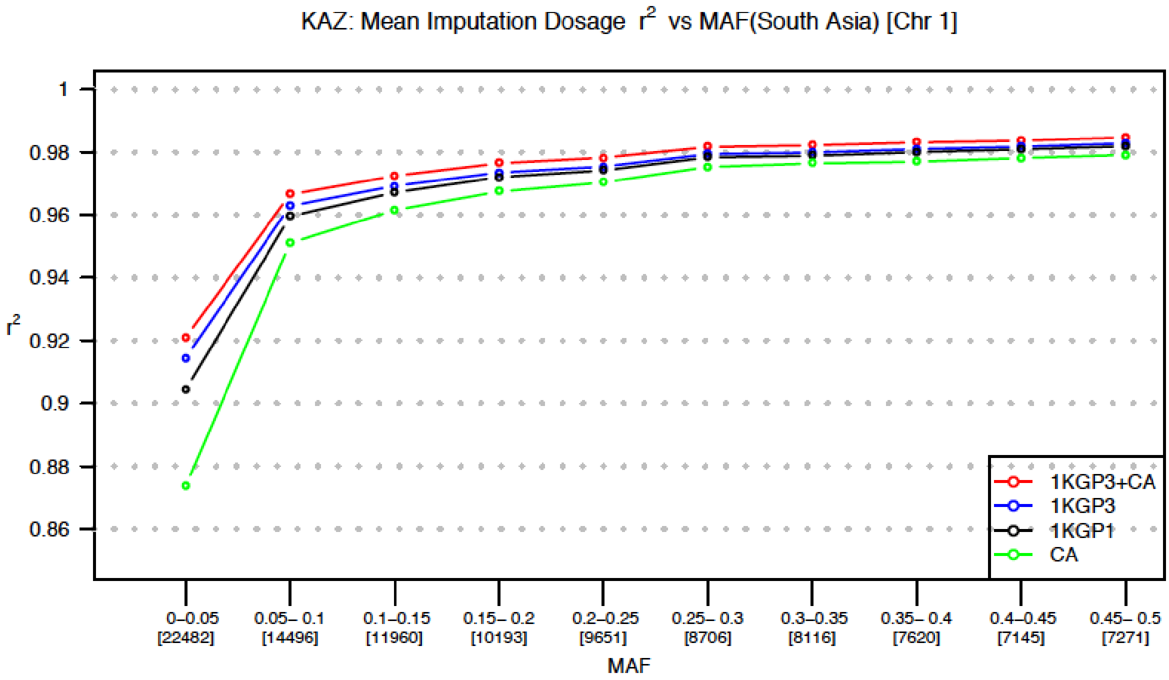


**Supplementary Figure 14: Imputation performance for Chromosome 1 vs South Asia MAF**
The Minor Allele Frequency (MAF) is as calculated in the 1000 Genomes Phase 3 Southern Asian samples. The number of variants in each MAF bin is shown underneath the label in square brackets.

**Supplementary Table 1 | Studies included in meta-analyses, follow-up and downstream analyses**

| Study | N cases | N controls | Population | Controls | Genotyping platform | Imputation reference set |
|---|---|---|---|---|---|---|
| ***Discovery stage*** | | | | | | |
| **Offspring meta-analysis** | | | | | | |
| GOPEC | 1,004 | 5,286 | UK | Population | Cases: Illumina OmniExpress; Controls: Illumina 1M | 1000G P1 |
| deCODE | 2,221 | 360,005 | Iceland | Population | Illumina (various) | deCODE WGS |
| ALSPAC | 146 | 6,130 | UK | Non PE offspring | Illumina HumanHap550 | 1000G P1 |
| MoBa | 1,046 | 961 | Norway | Non PE offspring | Illumina HumanCoreExome-12 | 1000G P3 |
| SSI | 213 | 963 | Denmark | Population | Illumina MEGA$^{EX}$ | HRC |
| Total Europe | 4,630 | 373,345 | | | | |
| Kazakh 1 | 789 | 795 | Kazakhstan | Non PE mothers | Illumina 2.5-8 | 1000G P3+ CA WGS |
| Kazakh 2 | 542 | 349 | Kazakhstan | Non PE mothers | Illumina OmniExpress | 1000G P3+ CA WGS |
| Uzbek | 814 | 883 | Uzbekistan | Non PE mothers | Illumina 2.5-8 | 1000G P3+ CA WGS |
| Total Central Asia | 2,145 | 2,027 | | | | |
| Europe + Central Asia | **6,775** | **375,372** | | | | |
| **Maternal meta-analysis** | | | | | | |
| GOPEC | 1,875 | 5,088 | UK | Population | Cases: Illumina 670; Controls: Illumina 1M | 1000G P1 |
| deCODE | 2,453 | 134,837 | Iceland | Female Population | Illumina (various) | deCODE WGS |
| ALSPAC | 150 | 6,161 | UK | Non PE mothers | Illumina human660W | 1000G P1 |
| MoBa | 1,469 | 954 | Norway | Non PE mothers | Illumina HumanCoreExome-12 | 1000G P3 |
| SSI | 872 | 815 | Denmark | Non PE mothers | Illumina MEGA$^{EX}$ | HRC |
| FINRISK | 400 | 7,805 | Finland | Non PE mothers | Illumina HumanCoreExome-12 | Finnish WGS/WES |
| Total Europe | 7,219 | 155,660 | | | | |
| Kazakh 1 | 846 | 814 | Kazakhstan | Non PE mothers | Illumina 2.5-8 | 1000G P3+ CA WGS |
| Kazakh 2 | 640 | 361 | Kazakhstan | Non PE mothers | Illumina OmniExpress | 1000G P3+ CA WGS |
| Uzbek | 810 | 884 | Uzbekistan | Non PE mothers | Illumina 2.5-8 | 1000G P3+ CA WGS |
| Total Central Asia | 2,296 | 2,059 | | | | |
| Europe + Central Asia | **9,515** | **157,719** | | | | |
| ***Follow up*** | | | | | | |
| **Offspring analysis** | | | | | | |
| FINNPEC | 580 | 760 | Finland | Non PE offspring | TaqMan | |
| Kazakh 3 | 452 | 361 | Kazakhstan | Non PE mothers | Illumina Infinium GSA | 1000G P3+ CA WGS |
| Total offspring | **1,032** | **1,121** | | | | |
| **Maternal analysis** | | | | | | |
| FINNPEC | 678 | 700 | Finland | Non PE mothers | Sequenom iPLEX | |
| DNBC | 335 | 1,054 | Denmark | Non PE mothers | Centaurus (Nanogen) | |
| HUNT | 1,030 | 4,264 | Norway | Female Population | Illumina (various) | HRC+ HUNT WGS |
| Kazakh 3 | 592 | 361 | Kazakhstan | Non PE mothers | Illumina Infinium GSA | 1000G P3+ CA WGS |
| Total maternal | **2,635** | **6,379** | | | | |
| ***Gestational hypertension*** | | | | | | |
| **Offspring analysis** | | | | | | |
| deCODE | 3,185 | 375,701 | Iceland | Population | Illumina (various) | deCODE WGS |
| ALSPAC | 1,090 | 6,039 | UK | Non PE/GH offspring | Illumina HumanHap550 | 1000G P1 |
| **Total offspring gestational hypertension** | **4,275** | **381,740** | | | | |
| **Maternal analysis** | | | | | | |
| deCODE | 2,380 | 133,925 | Iceland | Female Population | Illumina (various) | deCODE WGS |
| ALSPAC | 1,048 | 6,162 | UK | Non PE/GH mothers | Illumina HumanHap550 | HRC |

| | | |
|---|---|---|
| **Total maternal gestational hypertension** | **3,428** | **140,087** |

**Supplementary Table 2 |Follow up of variants with *P* < 1E-6 in the offspring meta-analysis**

| | | | | | | Discovery | | FINNPEC N=580 cases/760 ctrl | | Kazakh 3 N=452 cases/361 ctrl | | Combined discovery and follow-up | | |
|---|---|---|---|---|---|---|---|---|---|---|---|---|---|---|
| Variant | Chr | Position | Risk allele | Other allele | RAF | OR (95%CI) | *P*-value | OR (95%CI) | *P*-value | OR (95%CI) | *P*-value | OR (95%CI) | *P*-value | Annotated gene/cytoband |
| rs4769612[a] | 13 | 29,138,498 | c | t | 0.52 | 1.17 (1.12-1.23) | 3.0E-11 | 1.27 (1.09-1.49) | 2.5E-03 | 1.27 (1.03-1.56) | 0.028 | 1.19 (1.13-1.24) | 4.3E-14 | *FLT1/13q12* |
| rs11614652 | 12 | 26,958,002 | g | c | 0.21 | 1.18 (1.10-1.25) | 2.2E-07 | | | 1.12 (0.81-1.54) | 0.50 | 1.18 (1.10-1.23) | 1.7E-07 | *ITPR2/12p11* |
| rs5866671 | 5 | 24,557,312 | t | ta | 0.20 | 1.18 (1.11-1.25) | 1.3E-07 | | | 1.02 (0.76-1.37) | 0.88 | 1.17 (1.10-1.25) | 2.0E-07 | *CDH10/5p14* |
| rs75293382 | 2 | 228,510,336 | c | t | 0.06 | 1.33 (1.19-1.49) | 6.2E-07 | | | 1.05 (0.74-1.47) | 0.80 | 1.30 (1.18-1.45) | 1.4E-06 | *C2orf83/2q36* |

Positions are based on Human genome version 19 (hg19), build 37. Chr, chromosome; RAF, risk allele frequency; OR, odds ratio; CI, confidence interval.

[a]FINNPEC: rs4769613 ($r^2$ = 1 with rs4769612)

**Supplementary Table 3 |Conditional analysis chr13 locus**

| | | | | Discovery | | | | Conditioned on rs4769612 | | Conditioned on rs4769612 and rs9508092 | |
|---|---|---|---|---|---|---|---|---|---|---|---|
| Variant | Chr | Position | RA/OA | $RAF_{EUR}$ | $RAF_{CA}$ | OR (95%CI) | *P* | OR (95%CI) | *P* | OR (95%CI) | *P* |
| rs4769612 | 13 | 29138498 | C/T | 0.52 | 0.47 | 1.17 (1.12-1.23) | 3.0E-11 | | | | |
| rs9508092 | 13 | 29186162 | T/C | 0.77 | 0.74 | 1.19 (1.12-1.26) | 1.6E-08 | 1.20 (1.13-1.27) | 3.0E-09 | | |
| rs71433277 | 13 | 29218967 | T/C | 0.15 | 0.25 | 1.19 (1.12-1.26) | 1.7E-08 | | | 1.15 (1.08-1.22) | 6.6E-06 |

Positions are based on Human genome version 19 (hg19), build 37. Chr, chromosome; RA, risk allele; OA, other allele; $RAF_{EUR,}$ risk allele frequency in European meta-analysis; $RAF_{CA,}$ risk allele frequency in Central Asian meta-analysis; OR, odds ratio; CI, confidence interval.

**Supplementary Table 4 | Correlation ($r^2$) between variants at the chr13 *FLT1* locus reported in this study and previous report [PMID: 28628106]**

| Variant | rs149427560 | rs4769612 | rs4769613 | rs9508092 | rs71433277 | rs12050029 |
|---|---|---|---|---|---|---|
| rs149427560 | **1** | 0 | 0 | 0.01 | 0 | 0 |
| rs4769612 | 0 | **1** | **0.99** | 0.00 | 0.00 | 0.00 |
| rs4769613 | 0 | **0.99** | **1** | 0.00 | 0.00 | 0.00 |
| rs9508092 | 0.01 | 0.00 | 0.00 | **1** | 0.01 | 0.02 |
| rs71433277 | 0 | 0.00 | 0.00 | 0.01 | **1** | **0.97** |
| rs12050029 | 0 | 0.00 | 0.00 | 0.02 | **0.97** | **1** |

**Supplementary Table 5 | Correlation in European and Kazakh samples between variants with the lowest *P*-value in the maternal preeclampsia meta-analysis and blood pressure variants reported in Evangelou et al. 2018 [PMID: 30224653]**

| Preeclampsia variant | Chromosome | Position hg19 | Blood pressure variant | Position hg19 | $r^2$ Europe | $r^2$ Kazakh |
|---|---|---|---|---|---|---|
| rs1918975 | 3 | 169179876 | rs419076 | 169100886 | 0.36 | 0.12 |
| rs1458038 | 4 | 81164723 | rs16998073 | 81184341 | 0.94 | 0.83 |
| rs10774624 | 12 | 111833788 | rs3184504 | 111884608 | 0.88 | 0.97 |
| rs259983 | 20 | 57735457 | rs6015450 | 57751117 | 0.74 | 0.60 |

**Supplementary Table 6 | PE or GH risk allele concordance with high BP allele at 892 other known BP variants**

| GWAS Meta-analysis | Total Available Variants [N] | Observed Concordant Variants [C] | Null hypothesis probability of observing [C] or more Concordant Variants[a] |
|---|---|---|---|
| European PE mothers | 892 | 588 | $P < 1.2E-21$ |
| Central Asian PE mothers | 883 | 504 | $P < 2.9E-05$ |
| European and Central Asian PE mothers | 891 | 591 | $P < 1.1E-22$ |
| | | | |
| European GH mothers | 888 | 564 | $P < 7.1E-16$ |

892 BP variants omit our genome-wide significant PE variants from list of 896 known BP variants (Supplementary Table 18 in Evangelou et *al*. 2018 [PMID: 30224653])

[a]Null hypothesis is *none* of 892 variants are associated with PE/GH; null rejected as improbable by very low p-values as calculated by two-tail binomial test of proportion.

PE, preeclampsia; GH, gestational hypertension

**Supplementary Table 7 | Results for associated variants in offspring and maternal and discovery meta-analyses**

| | | | | | | Offspring discovery meta-analysis | | Maternal discovery meta-analysis | | |
|---|---|---|---|---|---|---|---|---|---|---|
| Variant | Chr | Position | RA | OA | RAF | *P-value* | OR (95%CI) | *P-value* | OR (95%CI) | Annotated gene/cytoband |
| Fetal | | | | | | | | | | |
| rs4769612 | 13 | 29,138,498 | c | t | 0.52 | 3.0E-11 | 1.17 (1.12-1.23) | 8.9E-07 | 1.10 (1.06-1.14) | *FLT1/13q12* |
| Maternal | | | | | | | | | | |
| rs1918975 | 3 | 169,179,876 | t | c | 0.60 | 0.045 | 1.05 (1.00-1.10) | 2.5E-08 | 1.12 (1.07-1.16) | *MECOM/3q26* |
| rs1458038 | 4 | 81,164,723 | t | c | 0.33 | 6.0E-05 | 1.11 (1.05-1.16) | 4.2E-08 | 1.12 (1.07-1.16) | *FGF5/4q21* |
| rs10774624 | 12 | 111,833,788 | g | a | 0.40 | 0.28 | 1.03 (0.98-1.08) | 7.0E-08 | 1.12 (1.07-1.16) | *SH2B3/12q24* |
| rs1421085 | 16 | 53,800,954 | c | t | 0.39 | 1.4E-03 | 1.08 (1.03-1.14) | 2.0E-09 | 1.13 (1.08-1.17) | *FTO/16q12* |
| rs259983 | 20 | 57,735,457 | c | a | 0.14 | 9.4E-03 | 1.10 (1.02-1.18) | 2.2E-07 | 1.15 (1.09-1.22) | *ZNF831/20q13* |

Positions are based on Human genome version 19 (hg19), build 37. Chr, chromosome; RA, risk allele; OA, other allele; RAF, risk allele frequency; OR, odds ratio; CI, confidence interval.

**Supplementary Table 8 | Samples included in preeclampsia subgroup analysis**

| | N Offspring analysis | | | N Maternal analysis | | |
|---|---|---|---|---|---|---|
| | EO cases | LO cases | Controls | EO cases | LO cases | Controls |
| GOPEC | 250 | 653 | 5,286 | 454 | 1,226 | 5,088 |
| deCODE* | 380 | 1,183 | 372,012/371,209 | 393 | 1,223 | 136,400/137,522 |
| MoBa | 170 | 824 | 887 | 297 | 1,089 | 930 |
| SSI | | | | 653 | 219 | 815 |
| Total | 800 | 2,660 | 378,185 | 1,797 | 3,757 | 144,355 |

EO, early onset preeclampsia; LO, late onset preeclampsia

*The number of controls is reported for the early onset and late onset analysis respectively

**Supplementary Table 9 | Heritability of preeclampsia**

***Per Cohort Heritability***

| Region | Type | Cohort | Heritability (SE) | $P_H$ | N | % Cases |
|---|---|---|---|---|---|---|
| Europe | Maternal | GOPEC | 0.420 (0.050) | 8.9E-17 | 6,971 | 26.9% |
| Europe | Maternal | MoBa | 0.192 (0.111) | 0.082 | 2,270 | 59.6% |
| Europe | Maternal | FINRISK | 0.378 (0.187) | 0.044 | 7,090 | 4.8% |
| Central Asia | Maternal | Uzbekistan | 0.699 (0.195) | 0.00034 | 1,738 | 51.6% |
| Central Asia | Maternal | Kazakhstan | 0.432 (0.166) | 0.0093 | 1,713 | 49.8% |
| Europe | Fetal | GOPEC | 0.193 (0.084) | 0.022 | 6,266 | 16.0% |
| Europe | Fetal | MoBa | 0.266 (0.134) | 0.048 | 1,864 | 53.0% |
| Central Asia | Fetal | Uzbekistan | 0.572 (0.201) | 0.0044 | 1,702 | 50.4% |
| Central Asia | Fetal | Kazakhstan | 0.323 (0.167) | 0.053 | 1,622 | 48.4% |

***Fixed effect meta-analysis***

| Region | Type | Heritability (95% CI) | *P* | $P_{het}$ | $I^2$ |
|---|---|---|---|---|---|
| Europe | Maternal | 0.381 (0.293,0.468) | 1.4E-17 | 0.17 | 42.6 |
| Europe | Fetal | 0.213 (0.074,0.353) | 0.0027 | 0.64 | 0 |
| Central Asia | Maternal | 0.544 (0.296,0.793) | 1.7E-05 | 0.30 | 7.5 |
| Central Asia | Fetal | 0.425 (0.173,0.676) | 0.00094 | 0.34 | 0 |
| Combined | Maternal | 0.399 (0.316,0.481) | 2.6E-21 | 0.22 | 32.7 |
| Combined | Fetal | 0.263 (0.141,0.384) | 2.4E-05 | 0.15 | 52.1 |

SE, standard error. $P_H$, P-Values are for a two-sided test that heritability differs from zero assuming that the GCTA heritability estimate is normally distributed with the specified standard error. CI, confidence interval. P, P-values are two sided and derived from fixed-effect meta-analysis of heritability.

**Supplementary Table 10 | Effect of preeclampsia variants on gestational hypertension**

| | | | | | Preeclampsia | | Gestational hypertension | | |
|---|---|---|---|---|---|---|---|---|---|
| Variant | Chr | Position | EA/OA | $EAF_{EUR}$ | OR (95%CI) | *P* | OR (95%CI) | *P* | Annotated gene/cytoband |
| Offspring meta-analysis[a] | | | | | | | | | |
| rs4769612 | 13 | 29,138,498 | C/T | 0.52 | 1.19 (1.13-1.24) | 4.3E-14 | 1.04 (0.98-1.11) | 0.21 | *FLT1/13q12* |
| Maternal meta-analysis[b] | | | | | | | | | |
| rs1918975 | 3 | 169,179,876 | T/C | 0.60 | 1.10 (1.07-1.14) | 1.2E-08 | 1.02 (0.97-1.08) | 0.46 | *MECOM/3q26* |
| rs1458038 | 4 | 81,164,723 | T/C | 0.33 | 1.11 (1.07-1.15) | 1.2E-08 | 1.06 (0.99-1.13) | 0.077 | *FGF5/4q21* |
| rs1421085 | 16 | 53,800,954 | C/T | 0.39 | 1.11 (1.07-1.15) | 1.2E-09 | 1.09 (1.03-1.15) | 1.7E-03 | *FTO/16q12* |
| rs259983 | 20 | 57,735,457 | C/A | 0.14 | 1.17 (1.11-1.23) | 2.9E-10 | 1.05 (0.97-1.14) | 0.22 | *ZNF831/20q13* |
| rs10774624 | 12 | 111,833,788 | G/A | 0.40 | 1.11 (1.07-1.15) | 1.7E-08 | 1.13 (1.07-1.20) | 3.7E-05 | *SH2B3/12q24* |

Positions are based on Human genome version 19 (hg19), build 37. Chr, chromosome; EA, effect allele; OA, other allele; $EAF_{EUR,}$ risk allele frequency in European meta-analysis; OR, odds ratio; CI, confidence interval.

[a]Association of fetal PE variant was tested in 4,275 offspring of gestational hypertension pregnancies and 381,740 controls

[b]Maternal variants were tested in 3,428 gestational hypertension cases and 140,087 controls

**Supplementary Table 11| Genetic correlation between maternal preeclampsia and selected secondary traits**

| | Samples included in secondary trait analysis in deCODE data | | Genetic correlation with maternal preeclampsia meta-analysis of GOPEC, ALSPAC and MoBa data | | | Samples included in secondary trait analysis in UKBB data | | Genetic correlation with maternal preeclampsia meta-analysis of deCODE, SSI and FINRISK data[*] | | | Combined genetic correlation | | |
|---|---|---|---|---|---|---|---|---|---|---|---|---|---|
| Secondary trait | N cases | N ctrl | $r_g$ | SE | *P*-value | N cases | N ctrl | $r_g$ | SE | *P*-value | meta $r_g$ | SE | *P*-value |
| Diastolic blood pressure | 127,761 | NA | 0.49 | 0.116 | 2.4E-05 | 385,919 | NA | 0.36 | 0.079 | 3.7E-06 | 0.40 | 0.065 | 5.6E-10 |
| Systolic blood pressure | 127,767 | NA | 0.37 | 0.100 | 2.0E-04 | 385,916 | NA | 0.33 | 0.069 | 2.2E-06 | 0.34 | 0.057 | 1.8E-09 |
| Hypertension | 54,974 | 324,803 | 0.48 | 0.127 | 2.0E-04 | 77,566 | 331,087 | 0.37 | 0.079 | 3.0E-06 | 0.40 | 0.067 | 3.1E-09 |
| Coronary artery disease | 37,878 | 320,415 | 0.17 | 0.123 | 0.16 | 10,860 | 397,707 | 0.37 | 0.090 | 4.4E-05 | 0.30 | 0.073 | 3.6E-05 |
| Ischemic stroke | 9,878 | 369,026 | 0.00 | 0.250 | 1.00 | 3,299 | 405,268 | 0.26 | 0.323 | 0.43 | 0.10 | 0.208 | 0.63 |
| Venous Thromboembolism | 4,967 | 343,276 | 0.45 | 0.190 | 1.8E-02 | 6,956 | 401,611 | 0.00 | 0.179 | 0.98 | 0.21 | 0.131 | 0.11 |
| Chronic kidney disease | 3,870 | 358,181 | 0.27 | 0.394 | 0.49 | 4,537 | 404,030 | 0.03 | 0.191 | 0.89 | 0.07 | 0.164 | 0.67 |
| Type 2 diabetes | 11,448 | 278,376 | 0.43 | 0.118 | 3.0E-04 | 22,389 | 386,178 | 0.29 | 0.078 | 2.0E-04 | 0.33 | 0.065 | 3.4E-07 |
| Body mass index (BMI) | 82,556 | NA | 0.12 | 0.082 | 0.13 | 407,412 | NA | 0.13 | 0.056 | 2.1E-02 | 0.13 | 0.047 | 5.9E-03 |
| Birth weight of first child | NA | NA | NA | NA | NA | 178,241 | NA | -0.28 | 0.074 | 2.0E-04 | -0.28 | 0.074 | 2.0E-04 |
| Asthma | 16,307 | 351,734 | -0.18 | 0.168 | 0.29 | 52,942 | 355,713 | -0.05 | 0.071 | 0.46 | -0.07 | 0.065 | 0.28 |
| Rheumatoid Arthritis | 2,201 | 339,871 | -0.01 | 0.318 | 0.98 | 4,047 | 404,518 | 0.09 | 0.183 | 0.64 | 0.06 | 0.150 | 0.69 |

[*]Analysis of genetic correlation between birth weight and preeclampsia also includes MoBa data

$r_g$, genetic correlation; SE, standard error; NA, not applicable

**Supplementary Table 12 | Polygenic risk score analysis**

| | Preeclampsia (1,703 cases and 74,678 controls) | | | | | | Gestational hypertension (1,553 cases and 75,109 controls) | | | | | |
|---|---|---|---|---|---|---|---|---|---|---|---|---|
| | | | | Adjusted for HT-PRS | | | | | | Adjusted for HT-PRS | | |
| PRS phenotype | *P* | effect | 95% CI | *P* | effect | 95% CI | *P* | effect | 95% CI | *P* | effect | 95% CI |
| Hypertension | 1.2E-12 | 0.18 | (0.13, 0.23) | | | | 2.1E-35 | 0.32 | (0.27, 0.38) | | | |
| Type 2 diabetes | 3.2E-03 | 0.07 | (0.02, 0.12) | 0.032 | 0.05 | (0.00, 0.10) | 0.16 | 0.04 | (-0.01, 0.09) | 1.00 | 0 | (-0.05, 0.05) |
| Body mass index | 6.1E-03 | 0.07 | (0.02, 0.12) | 0.099 | 0.04 | (-0.01, 0.09) | 2.5E-05 | 0.11 | (0.06, 0.16) | 0.022 | 0.06 | (0.01, 0.11) |
| Coronary artery disease | 4.1E-03 | 0.07 | (0.02, 0.12) | 0.091 | 0.04 | (-0.01, 0.09) | 3.9E-03 | 0.08 | (0.02, 0.13) | 0.44 | 0.02 | (-0.03, 0.07) |
| Birth weight | 1.5E-05 | -0.11 | (-0.16, -0.06) | 5.0E-04 | -0.09 | (-0.14, -0.04) | 8.3E-05 | -0.10 | (-0.15, -0.05) | 0.017 | -0.06 | (-0.11, -0.01) |

**Supplementary Table 13 | Clinical characteristics**

| Cohort and country of origin | Group | Maternal age* | Primiparous pregnancies | BMI† | Highest systolic blood pressure* | Highest diastolic blood pressure* | Gestation at delivery (weeks)† | Offspring birthweight (grams)† |
|---|---|---|---|---|---|---|---|---|
| *Preeclampsia GWAS* | | | | | | | | |
| GOPEC# UK | Cases n=1875 | 28.7 (5.9) | 73% | 25.4 (22.6-29.5) | 166 (18) | 109 (10) | 37 (34-39) | 2608 (1863-3184) |
| ALSPAC UK | Cases n=146 | 28.7 (5.3) | 68% | 23.1 (21.3-27.5) | 159 (13) | 108 (9) | 39 (37-40) | 3240 (2710-3680) |
| ALSPAC UK | Controls n=6130 | 28.6 (4.8) | 41% | 21.9 (20.4-23.9) | 127 (10) | 78 (7) | 40 (39-41) | 3460 (3160-3780) |
| deCODE# Iceland | Cases n=2618 | 28.0 (6.3) | 66% | 25.0 (22.0-29.7) | 151 (15) | 102 (9) | 39 (37-40) | 3294 (2730-3712) |
| MoBa Norway | Cases n=3074 | 29.6 (4.9) | 63% | 24.8 (22.1-28.7) | 146 (14) | 95 (9) | 39 (37-40) | 3310 (2754-3760) |
| MoBa Norway | Controls n=1599 | 30.2 (4.5) | 43% | 23 (21.2-25.7) | 125 (11) | 78 (8) | 40 (40-41) | 3640 (3300-3990) |
| FINRISK Finland | Cases n=400 | 29.3 (6.0) | 64% | NA | NA | NA | 39 (36-40) | 2970 (2365-3545) |
| FINRISK Finland | Controls n=7805 | 27.7 (5.4) | 95% | NA | NA | NA | 40 (39-41) | 3460 (3150-3780) |
| SSI Denmark | Cases n=872 | 27.4 (4.8) | NA | 24.5 (21.7-29.4) | NA | NA | 35 (31-39) | 2010 (954-3066) |
| SSI Denmark | Controls n=815 | 26.5 (4.4) | NA | 23.5 (21.1-26.2) | NA | NA | 41 (39-43) | 3615 (3045-4185) |
| Kazakh Pregnancy Biobank Kazakhstan | Cases n=2602 | 26.7 (6.4) | 53% | 27.5 (24.3-31.2) | 160 (11) | 105 (7) | 37 (35-39) | 2840 (2151-3350) |
| Kazakh Pregnancy Biobank | Controls | 25.9 (5.6) | 53% | 25.7 | 119 (5) | 78 (5) | 40 | 3400 |

| | | | | | | | | |
|---|---|---|---|---|---|---|---|---|
| Khazakstan | n=2603 | | | (23.2-28.7) | | | (38-41) | (3060-3700) |
| Uzbek Pregnancy Biobank Uzbekistan | Cases n=2005 | 25.5 (4.9) | 68% | 24.3 (22.3-27.0) | 158 (12) | 108 (8) | 37 (35-39) | 2700 (2100-3200) |
| Uzbek Pregnancy Biobank Uzbekistan | Controls n=2006 | 24.4 (4.2) | 66% | 23.7 (21.7-25.8) | 108 (7) | 70 (6) | 39 (39-40) | 3380 (3100-3650) |
| *Gestational hypertension* | | | | | | | | |
| deCODE Iceland | Cases n=2814 | 29.4 (6.2) | 54% | 24.2 (21.5-28.2) | 143 (12) | 99 (8) | 40 (39-41) | 3620 (3280-3988) |
| ALSPAC UK | Cases n=1252 | 28.5 (5.0) | 58% | 23.5 (21.5-26.9) | 149 (12) | 95 (12) | 40 (39-41) | 3440 (3100-3780) |
| ALSPAC UK | Controls n=6130 | 28.6 (4.8) | 41% | 21.9 (20.4-23.9) | 127.1 (9.9) | 77.8 (7.0) | 40 (39-41) | 3460 (3160-3780) |
| *Follow-up datasets* | | | | | | | | |
| FINNPEC Finland | Cases n=678 | 30.2 (5.6) | 75% | 23.7 (21.3-27.1) | 166 (17) | 109 (8) | 39 (35-39) | 2825 (2180-3325) |
| FINNPEC Finland | Controls n=760 | 29.7 (5.0) | 54% | 23.0 (20.8-25.9) | 126 (12) | 83 (8) | 40 (39-41) | 3628 (3290-3960) |
| HUNT[#] Norway | Cases n=1134 | 26.5 (5.6) | 39% | 26.4 (23.7-29.6) | NA | NA | 40 (38-41) | 3330 (2760-3810) |
| DNBC Denmark | Cases n=335 | 28.7 (4.6) | NA | 24.8 (22.1-29.6) | NA | NA | 39 (37-41) | 3150 (2200-4100) |
| DNBC Denmark | Controls n=1050 | 27.7 (3.9) | NA | 23.3 (21.0-26.2) | NA | NA | 40 (39-41) | 3500 (2900-4100) |

*Mean and standard deviation

†median and interquartile range

[#]The GOPEC, deCODE and HUNT studies used population controls

NA: Not available

**Supplementary Table 14 | Secondary traits from deCODE and UKBB**

| | deCODE trait definition | UKBB trait definition* |
|---|---|---|
| Diastolic blood pressure | BP measurements were obtained from Landspitali - the National University Hospital of Iceland in Reykjavik (LUH), the Primary Health Care Clinics of the Reykjavik area and at recruitment for deCODE studies, totaling measurements for 145,615 individuals with an average of 12 measurements per individual. Measurements were adjusted for sex, year of birth, age at measurement, measurement center and for use of drugs (by adding 10 mmHg to measured values for those taking blood pressure lowering drugs). Blood pressure measurements were subsequently standardized to have a normal distribution. | Diastolic blood pressure measurements (field 4079) adjusted for age at measurement, sex, year of birth, 40 principle components and for use of drugs (by adding 10 mmHg to measured values for those taking blood pressure lowering drugs). The adjusted trait values were then standardized using an inverse normal transform. |
| Systolic blood pressure | BP measurements were obtained from Landspitali - the National University Hospital of Iceland in Reykjavik (LUH), the Primary Health Care Clinics of the Reykjavik area and at recruitment for deCODE studies, totaling measurements for 145,615 individuals with an average of 12 measurements per individual. Measurements were adjusted for sex, year of birth, age at measurement, measurement center and for use of drugs (by adding 15 mmHg to measured values for those taking blood pressure lowering drugs). Blood pressure measurements were subsequently standardized to have a normal distribution. | Systolic blood pressure measurements (field 4080) adjusted for age at measurement, sex, year of birth, 40 principle components and for use of drugs (by adding 15 mmHg to measured values for those taking blood pressure lowering drugs). The adjusted trait values were then standardized using an inverse normal transform. |
| Hypertension | ICD10 codes I10.x,I11.x,O10.x, O11.x, I15.x (or the corresponding ICD9 codes) obtained from Landspitali - the National University Hospital of Iceland in Reykjavik (LUH) and the Primary Health Care Clinics of the Reykjavik area. | ICD10 code I10 in primary or secondary hospital diagnoses codes (fields 41202 and 41204). |

| | | |
|---|---|---|
| Coronary artery disease | ICD10 codes I20.0, I21.x, I22.X, I24.x, I25.x (or the corresponding ICD9 codes) from LUH or the Icelandic death registry. | ICD10 codes I20.0, I21.x, I22.X, I24.x, I25.x, or operation proceedure codes indicative of CAD obtained from primary or secondary diagnoses codes a participant has had recorded across all their episodes in hospital. |
| Ischemic stroke | ICD9 codes 433.x,434.x,435.x,4371,438.x and ICD10 codes I63.x,I693x from LUH. | ICD10 codes I63.x and I69.3x obtained from primary or secondary diagnoses codes a participant has had recorded across all their episodes in hospital. |
| Venous Thromboembolism | ICD9 codes 415, 415.1, 451, 451.1, 451.2, 451.8, 451.9, 453.1, 453.8, 459.1 and ICD10 codes I26, I26.9, I80, I80.1, I80.2, I80.3, I80.9, I82.1, I82.8, 187.0 obtained from LUH. | ICD9 codes 415, 415.1, 451, 451.1, 451.2, 451.8, 451.9, 453.1, 453.8, 459.1 and ICD10 codes I26, I26.9, I80, I80.1, I80.2, I80.3, I80.9, I82.1, I82.8, 187.0 obtained from primary or secondary diagnoses codes a participant has had recorded across all their episodes in hospital. |
| Chronic kidney disease | ICD10 code N18 | ICD10 code N18 |
| Type 2 diabetes | History of type 2 diabetes (self reported); HbA1C > 6.5; clinical diagnosis; ICD10 code E11; use of oral diabetes medication. Subjects with type 1 diabetes were excluded from the study. | Type 2 diabetes based on ICD10 code E11 from primary or secondary hospital diagnosis (fields 41202 and 41204), on self-reported illnss codes 1220 and 1223 (field 20002) and on thouchscreen question on diabetes (field 2443). Individuals with diagnosis of gestational diabetes (ICD10 code O244, self-reported illness code 1221 or thouchscreen question field 4041) or with type 1 diabetes (based on early insulin use, field 2986, or self-reported illness code 1222) were excluded. |
| Body mass index (BMI) | BMI information was available for 86,518 Icelandic individuals (Thorleifsson et.al. 2009 [PMID: 19079260] after excluding individuals not of Icelandic origin. The measurements were adjusted for gender, age, age$^2$ and county of origin within Iceland, then averaged over multiple measurements for an individual, and standardized by using an inverse normal transform. | Body mass index (field 21001) adjusted for age at measurment, sex, year of birth and 40 principle components. The adjusted trait values were then standardized using an inverse normal transform. |
| Birth weight | NA | Self-reported birth weight of first child (field 2744) adjusted for year of birth and 40 principle components. The adjusted trait values were then standardized using an inverse normal transform. |

| | | |
|---|---|---|
| Asthma | Icelandic asthma patients over 18 years of age were recruited who attended an asthma clinic or emergency rooom at the National University Hospital of Iceland or the Icelandic Medical Center (Laeknasetrid) during the years 1977 to 2017. Asthma diagnosis was based on a combination of physician‘s diagnosis and ICD10 diagnosis, including anyone of J45.0, J45.1, J45.8, J45.9 and J46 and/or self-reported by a positive reply to the question: “Has a doctor confirmed your asthma diagnosis“. | Asthma in the UK Biobank was defined as ICD10 diagnoses in fields 41202 or 41204, including anyone of J45.0, J45.1, J45.8, J45.9 and J46 and/or self-reported by the non-cancer illness code, self-reported during verbal interview (data-field 20002) with a code for asthma (1111). |
| Rheumatoid Arthritis | Diagnosis of rheumatoid arthritis based on fulfillment of classification criteria (ACR 1987 criteria) and/or ICD10 codes M058, M059, M060, M068, M069 obtained from LUH or other rheumatology units in Iceland. | Diagnosis of rheumatoid arthritis based on ICD10 codes M058, M059, M060, M068, M069 obtained from UK Biobank. |

*We included only individuals who were self-reported white British with similar genetic ancestry based on principal component analysis and with consistently reported and genetically determined gender.

# Supplementary Notes

## Supplementary Note 1: Construction of Central Asia Haplotype Reference Panel

Extensive efforts have been made to provide reference panels of whole genome sequencing data from diverse populations through initiatives such as 1000Genomes, but Central Asian populations have not so far been included in these panels. Central Asia lies at the centre of the Silk Road, historic trading routes between Asia and Europe. The traditional nomadic lifestyle of much of the population, and repeated past invasions by surrounding powers, created a population of mixed ethnicity. During the Soviet era large numbers of Russians were resettled in Central Asia, and ethnic Russians made up a significant proportion of the population. The movement of ethnic Russians has reversed since the Central Asian republics gained independence in the early 1990s, and the two largest ethnic groups are now Kazakhs and Uzbeks.

Whilst reference panels for genotype imputation are readily available for European populations, no such panel was available for the Kazakh and Uzbek populations of Central Asia. We therefore undertook whole genome sequencing (WGS) of 100 Kazakh and 100 Uzbek individuals, equally divided between males and females, recruited from Kazakhstan and Uzbekistan respectively. The ancestry of each Central Asian volunteer was determined by the ethnicity of all four grandparents. Of interest, Kazakhs are represented by three hoards, or zhus – older, middle and younger – and information about grandparental zhus was recorded for each Kazakh subject.

*Construction of Central Asia Haplotype Reference Panel*

200 Central Asian individuals (100 Kazak and 100 Uzbek) were whole genome sequenced at a coverage of approximately 4-5X. Variant calling discovered 11,870,850 single nucleotide polymorphisms (SNP) and 1,013,884 indels including over 2 million variants not detected in 1000 Genomes Phase 1. Phased genotypes were used to create a haplotype reference panel, and genotype imputation performance was assessed in 1600 chip-genotyped subjects. Combining reference data from Central Asian WGS and 1000Genomes Phase 3 yielded better imputation quality than using ether reference panel alone.

*Population Structure*

A PCA analysis of the combined Central Asian WGS and 1000 Genomes Phase 3 (Europe, South Asia and East Asia) data indicates that the Kazakh and Uzbek populations are on a cline between East Asia and Europe with Uzbekistan exhibiting a greater affinity with Europe and South Asia (Supplementary Figure 6). Importantly, the analysis shows the two Central Asian populations clustering separately from any of the Eurasian 1KGP3 populations.

*Imputation Quality*

We assessed the imputation quality using the internal IMPUTE2 leave one-out measurements of the squared correlation, $r^2$, between the genotype dosage of directly genotyped variants vs the expected dosage of the corresponding imputed variants. We randomly selected 800 unrelated GWAS samples from each population and pre-phased each grouping of samples separately. The phased samples were then imputed into 4 different panels; 1000 Genomes Phase 3 +

Central Asia (1KGP3+CA), 1000 Genomes Phase 3 (1KGP3), 1000 Genomes Phase 1 (1KGP1) and Central Asia (CA). The imputation quality was assessed at sites that are present on both the chip and the intersection of tested reference panels. We find that both Uzbek and Kazakh samples exhibit better imputation quality across the full range of Central Asia allele frequencies with the combined panel (1KGP3+CA) than with any of the other panels (Supplementary Figure 7). Performance relative to allele frequencies calculated in other regions exhibit a similar pattern (Supplementary Figures 12, 13 and 14).

## *Methods*

### *Ethics Statement*

This study was approved by the Central Commission on Ethics of the Republic of Kazakhstan, the National Ethics Committee of the Ministry of Health of the Republic of Uzbekistan, and the Medical School Research Ethics Committee of the University of Nottingham. Volunteers in each Central Asian country gave informed consent and provided an irreversibly anonymised sample of venous blood for DNA extraction.

### *Subject recruitment and DNA Isolation*

200 Kazakh and 200 Uzbek subjects for WGS were recruited from healthy volunteers in Almaty, Kazakhstan, and Tashkent, Uzbekistan respectively. Only 100 of the 200 subjects from each country were selected for whole genome sequencing in order to enhance subject anonymity and protect the identity of the subjects whose genomes were sequenced.

The grandparental ethnicity of all four grandparents of each subject was recorded to minimise ethnic admixture. Kazakhs belong to one of three Zhus (hordes); the Zhu of each subject was recorded at the time of recruitment and subjects were selected for WGS to account for the approximate composition of Zhus in Kazakhstan. There were no corresponding ethnic strata in the Uzbek subjects who were therefore randomly selected. Subjects selected for WGS were equally split between males and females for each country. DNA was extracted in the country of origin and transferred to the Wellcome Trust Sanger Institute, UK, where it was subjected to quality control measures prior to sequencing. These included measurement of DNA concentration both by absorbance at 260nm/280nm and by the pico green method; gel electrophoresis to check for DNA degradation, and Sequenom genotyping at 30 SNPs, including four sex-specific variants. Samples which failed gender checks, or where Sequenom genotyping was unsuccessful at 10 or more SNPs, were not selected for WGS.

### *Library Preparation and Sequencing*

Approximately 1ug genomic DNA for each subject was fragmented to an average size of 500 base pairs (bp) and subjected to DNA library creation using established Illumina paired-end protocols. Adapter-ligated libraries were amplified and indexed via PCR. A portion of each library was used to create an equimolar pool comprising 8 indexed libraries. Libraries were subjected to 100 base paired-end sequencing (HiSeq 2000; Illumina) according to manufacturer instructions.

### *Alignment and assembly of short reads into whole genome sequence*

For each subject, several million short (~100 base) paired-end reads generated by Illumina HiSeq 2000 sequencer were aligned with human reference sequence using the Sanger Institute sequencing pipeline

originally created for the 1000 Genomes Project (1000 Genomes Project Consortium 2010, 2012). Briefly, this involved the following steps:

- Aligned to reference (1000 genomes hs37d5) using BWA (bwa-0.5.10-mt)
- Duplicates removed using Picard MarkDuplicates (picard-tools-1.72)
- Merged to sample level BAMs
- Realignment around known indels GATK RealignerTargetCreator and IndelRealigner
- GATK BQSR (Base Quality Score Realignment)
- Called using:
    - samtools-0.1.19 mpileup -EDVSp -C50 -m3 -F0.2 -d 40
    - samtools-0.1.19/bcftools/bcftools view -m 0.99 -vcgN
- Variant filtering was carried out using VQSR GenomeAnalysisTK-2.7-2

*Construction of haplotype reference panels*

The Central Asia panel was created from the above filtered variants as follows:

- The novel variants (i.e. those not in 1000 Genomes Phase 3 (1KGP3)) were merged with 1KGP3 Biallelic and Multiallelic sites.

- All variants were recalled using:
  GATK UnifiedGenotyper --genotyping_mode GENOTYPE_GIVEN_ALLELES.

- The resulting genotype likelihoods were processed as follows:

- An initial genotype refinement for 1KGP3 variants was carried out with Beagle (v4 (r1399.jar)) using the 1KGP3 panel (Europe+East Asia+South Asia groups only) downloaded from the Beagle website.

- The genotype probabilities generated by this initial genotype refinement were then fixed as hard-called genotypes and merged with the genotype likelihoods of novel variants.

- Beagle was then run again without a reference panel on this merged data-set in order to obtain refined calls for the novel variants.

- The refined genotype probabilities for the novel variants were then extracted and merged with the refined genotype probabilities for 1KGP3 variants obtained from the first run of Beagle.

- The Beagle vcf files were converted into Oxford gen format and then hard-called and filtered using plink2 (--hard-call-threshold 0.1 --geno 0.05) and saved again in Oxford gen format.

- Shapeit (v2.r837) was then run with default parameters (again with no reference panel).

The Central Asia + 1000 Genomes Phase 3 Reference Panel (1KGP3+CA) was constructed in 5Mb chunks using impute2 (-merge -Ne 20000 -buffer 500 -k_hap 1600).

*Concordance of Chip Genotypes*

The WGS samples were also chip genotyped; 69 samples on Illumina OmniExpress 2.5.8 and 134 samples on Illumina OmniExpress (some samples were genotyped on both platforms). Basic QC was carried out on these chip genotypes (call rate>98% and heterozygosity within 3 s.d. of the mean). Supplementary Figures 10 and 11 demonstrate the accuracy of the refined genotype probabilities.

*Imputation Benchmarking*

The chip genotyped samples from Uzbekistan and Kazakhstan were separately QC'd using the following protocol. Quality control analysis was conducted using PLINK (http://zzz.bwh.harvard.edu/plink/) and SMARTPCA. Briefly, the quality control included the following subject-level exclusion criteria: individual call rate <98%, heterozygosity >3 s.d. from the mean; any of the first three HapMap (based on CEU, YRI, CHB, JPT and GIH populations) principal axes of variation >4 s.d. from the mean; and sex mismatch. Related individuals (identity by descent (IBD) > 0.1) with the lowest call rates were preferentially removed. The variant-level exclusion criteria were as follows: call rate <98%; exact Hardy–Weinberg equilibrium P < 1 × 10−6; minor allele frequency (MAF) <1%; and non-random missingness of uncalled genotypes (plink–test-mishap) with Bonferroni-corrected P < 0.05.

From each of the above datasets 800 female samples that passed the above subject-level QC were selected and 123,250 chromosome 1 chip genotyped SNPs that were present in both KAZ and UZB post QC datasets.

The samples were pre-phased without a reference panel using shapeit2 (v2.r727) with default parameters except ( --effective-size 12000). The samples were then imputed with impute2 (v2.3.1) with default parameters except ( -buffer 500 -k_hap 2000).

*PCA Analysis*

The combined reference panel was first converted to VCF format using "bcftools convert -H" and then converted to plink format. The resulting variants were filtered to include variants with MAF>0.05 and pruned so that no variants within a window of 1Mb exhibit a pairwise LD $r^2 > 0.1$ (plink --indep-pairwise 1000kb 100 0.1). The first 10 Principal Components were then calculated using (plink –pca 10).

# Supplementary Note 2: Correcting for Population Stratification in EMIM

## *Introduction*

In [1] a method is introduced for inferring Maternal, Fetal, Imprinting and Maternal-Fetal Interaction effects from family genotype data. This method, EMIM, fits a genetic model using a multinomial likelihood model for the possible family genotype combinations constrained by Mendelian inheritance. Taking advantage of recent improvements in statistical haplotype phasing improvements a recent extension to the method [2] resolves heterozygous genotype cells to improve inference of parent of origin effects.

One difficulty in using EMIM in mildly heterogeneous populations is that it does not provide a means to control for population stratification. Here we present an extension that incorporates cohort indicator variables and ancestry principal components as covariates into the method. We provide an R package implementation of the method (remim).

## *Methods*

Following the notation in [1] we assume the following model for penetrance. Let A2 be the risk allele with allele frequency $A_2$ (alternate allele A1 has allele frequency $A_1$ = 1-$A_2$)

- $i$ copies of A2 in the child multiplies penetrance by $R_i$
- $i$ copies of A2 in the mother multiplies penetrance by $S_i$
- $i$ paternally transmitted copies A2 multiplies penetrance by $Ip_i$
- $i$ maternally transmitted copy A2 multiplies penetrance by $Im_i$
- $i$ maternal copies of A2 and j fetal copies A2 multiplies penetrance by $\gamma_{i,j}$

This gives the following equation for the penetrance:

$$p(D|mU, mT, pT, \boldsymbol{\theta}) = \alpha R_{mT+pT} S_{mU+mT} Im_{mT} Ip_{pT} \gamma_{mT+mU,pT+pU} \quad (1)$$

Where $\boldsymbol{\theta} = \{R_i, S_i, Ip_i, Im_i, \gamma_{i,j}, A_2\}$ and $mU, mT$ and $pT$ are the counts of the maternal untransmitted, maternal transmitted and paternal transmitted risk alleles and $\alpha$ is the disease prevalence.

The central equation in [1] is:

$$p(mU, mT, pT|D) = \frac{p(D|mU,mT,pT)}{\sum_{mU,mT,pT} p(D|mU,mT,pT)} p(mU, mT, pT) \quad (2)$$

Here we assume random mating, so that we can write:

$$p(mU, mT, pT) = {A_1}^{mU+mT+pT}(1 - A_1)^{3-(U+mT+pT)} \quad (3)$$

In the case where we have genotype data but are unable to infer transmitted and untransmitted alleles (e.g for sparsely genotyped datasets) it is necessary to sum over values of $mU, mT$ and $pT$ that are consistent with the genotype data:

$$p(gm, gc|D) = \sum_{mU,mT,pT:mU+mT=gm,mT+pT=gc} p(mU, mT, pT|D) \qquad (4)$$

$$p(gm, gc, gf|D) = \sum_{mU,mT,pT:mU+mT=gm,mT+pT=gc,pT+pU=gf} p(mU, mT, pT|D)\, p(pU) \qquad (5)$$

In practice it is necessary to partition the samples into maximal family units of various types: Case Trios, Case Duos, Case Mothers, Case Fathers, Case Offspring, Control Parents, Control Duos and Controls. Summing over these independent family units gives the following equation for the log likelihood of the observed allele counts/genotypes:

$$\begin{aligned}\mathcal{L}(\boldsymbol{mU}, \boldsymbol{mT}, \boldsymbol{pT}, \boldsymbol{pU}, \boldsymbol{gc}, \boldsymbol{gq}, \boldsymbol{gm}|\theta) = &\sum_{Case\ Trios} \log p(mU, mT, pT, pU|D, \boldsymbol{\theta}) \\ &+ \sum_{Case\ Duos} \log p(mU, mT, pT|D, \boldsymbol{\theta}) \\ &+ \sum_{Case\ Offspring} \log p(gc|D, \boldsymbol{\theta}) \\ &+ \sum_{Case\ Mothers} \log p(gm|D, \boldsymbol{\theta}) \\ &+ \sum_{Controls} \log p(gq|D, \boldsymbol{\theta})\end{aligned} \qquad (6)$$

The parameters $\boldsymbol{\theta}$ are then fitted by maximising the above likelihood and statistical significance is assessed by likelihood ratio tests on nested models or by estimating standard errors in the usual way using the inverse Hessian matrix. Note that the above model fits a single population allele frequency in the above process.

Here, rather than fitting a single allele frequency, we propose modelling individual specific genotype/allele probabilities (allele frequency) using a logit transform of the weighted sum of continuous covariates:

$$p(mT = 1) = p(mU = 1) = \frac{e^{\sum_{i=1}^{n} c_i^{(m)} \beta_i}}{1 + e^{\sum_{i=1}^{n} c_i^{(m)} \beta_i}} \qquad (7)$$

$$p(pT = 1) = p(pU = 1) = \frac{e^{\sum_{i=1}^{n} c_i^{(p)} \beta_i}}{1 + e^{\sum_{i=1}^{n} c_i^{(p)} \beta_i}} \qquad (8)$$

Where $c_i^{(m)}$ and $c_i^{(p)}$ are maternal and paternal covariates respectively (e.g. principal components or cohort indicator variable). In the case of Duos the missing parent covariate can be estimated by assuming that the child covariates $c_i^{(c)}$ (available by assumption) are the average of parental covariates. For lone

cases (Mothers, Fathers or Offspring) we do not necessarily have values for the missing parents. Under these circumstances we are forced to either remove the samples or to make an assortative mating assumption and set unknown covariates to the same value as the sampled family member. For lone controls the covariates of the individual sample are sufficient.

We incorporated these new variables into $\theta$ as $\boldsymbol{\theta} = \{R_i, S_i, Ip_i, Im_i, \gamma_{i,j}\beta_i\}$ so that the β coefficients are jointly estimated with the parameters of the EMIM genetic model by maximising the composite log-likelihood for the set of parameters:

$$
\begin{aligned}
\mathcal{L}(\boldsymbol{mU}, \boldsymbol{mT}, \boldsymbol{pT}, \boldsymbol{pU}, \boldsymbol{gc}, \boldsymbol{gq}, \boldsymbol{gm} | \boldsymbol{\theta}, \boldsymbol{c}) = & \sum_{Case\ Trios} \log p(mU, mT, pT, pU | D, \boldsymbol{\theta}, \boldsymbol{c}) \\
& + \sum_{Case\ Duos} \log p(mU, mT, pT | D, \boldsymbol{\theta}, \boldsymbol{c}) \\
& + \sum_{Case\ Offspring} \log p(gc | D, \boldsymbol{\theta}, \boldsymbol{c}) \\
& + \sum_{Case\ Mothers} \log p(gm | D, \boldsymbol{\theta}, \boldsymbol{c}) \\
& + \sum_{Controls} \log p(gq | D, \boldsymbol{\theta}, \boldsymbol{c})
\end{aligned}
\tag{9}
$$

We now set out the formulae for the probabilities in the above.

For Mother-Child Duos we have:

$$p(D | mU, mT, pT, \boldsymbol{\theta}) = R_{mT+pT} S_{mU+mT} (\delta_{mT,0} + \delta_{mT,1} I_m)(\delta_{pT,0} + \delta_{pT,1} I_p) \gamma_{mT+mU, pT+pU} \tag{10}$$

$$p(mU, mT, pT | D, \boldsymbol{\theta}, \boldsymbol{c}) = p(D | mU, mT, pT, \boldsymbol{\theta}) \frac{\exp\left\{pT \sum_{i=1}^{n} c_i^{(p)} \beta_i\right\}}{\left(1 + \exp\left\{\sum_{i=1}^{n} c_i^{(p)} \beta_i\right\}\right)} \frac{\exp\left\{(mU + mT) \sum_{i=1}^{n} c_i^{(m)} \beta_i\right\}}{\left(1 + \exp\left\{\sum_{i=1}^{n} c_i^{(m)} \beta_i\right\}\right)^2} \tag{11}$$

For Trios we have:

$$p(mU, mT, pT, pU | D, \boldsymbol{\theta}, \boldsymbol{c}) = p(mU, mT, pT | D, \boldsymbol{\theta}, \boldsymbol{c}) \frac{\exp\left\{pU \sum_{i=1}^{n} c_i^{(p)} \beta_i\right\}}{\left(1 + \exp\left\{\sum_{i=1}^{n} c_i^{(p)} \beta_i\right\}\right)} \tag{12}$$

For a Control Trios we have:

$$p(mU, mT, pT, pU | D, \boldsymbol{\theta}, \boldsymbol{c}) = \frac{\exp\left\{(pU + pT) \sum_{i=1}^{n} c_i^{(p)} \beta_i\right\}}{\left(1 + \exp\left\{\sum_{i=1}^{n} c_i^{(p)} \beta_i\right\}\right)^2} \frac{\exp\left\{(mU + mT) \sum_{i=1}^{n} c_i^{(m)} \beta_i\right\}}{\left(1 + \exp\left\{\sum_{i=1}^{n} c_i^{(m)} \beta_i\right\}\right)^2} \tag{13}$$

In the case where we only have genotype data available we can marginalise over *mU*, *mT*, *pU* and *pT*. So, for Case Mothers we have:

$$p(gm|D,\boldsymbol{\theta},\boldsymbol{c}) = \sum_{pT}\sum_{\{mU,mT:mU+mT=gm\}} p(mU,mT,pT|D,\boldsymbol{\theta},\boldsymbol{c}) \quad (14)$$

For Case Offspring:

$$p(gc|D,\boldsymbol{\theta},\boldsymbol{c}) = \sum_{mU}\sum_{\{mT,pT:pT+mT=gc\}} p(mU,mT,pT|D,\boldsymbol{\theta},\boldsymbol{c}) \quad (15)$$

For Controls:

$$p(gq|D,\boldsymbol{\theta},\boldsymbol{c}) = \frac{\exp\{gq\sum_{i=1}^{n} c_i\beta_i\}}{\left(1+\exp\{\sum_{i=1}^{n} c_i\beta_i\}\right)^2} \quad (16)$$

Where $c_i$ represent the *i*th principal component/indicator variable of the control individual.

## *Discussion*

The extension to include cohort indicator variables and principal components extends the scope of the method allowing the joint analysis of multiple cohorts and/or genetically heterogeneous populations. The cost of considering continuous covariates is that individuals must be included separately in the likelihood equation rather than grouped into cells containing the same genotype combinations. This makes the approach less computationally efficient and so less appropriate for analysing whole genome data.

We provide a R based implementation (remim) where we have made of use of explicit expressions for both the likelihood and likelihood gradient to facilitate the rapid convergence of R's "optim" function using the method "L-BFGS-B". The likelihood and gradient functions are implemented in C.

## Supplementary Note 3: Study Acknowledgements and Funding

ALSPAC
We are extremely grateful to all of the families who took part in ALSPAC, the midwives for their help in recruiting them, and the whole ALSPAC team, which includes interviewers, computer and laboratory technicians, clerical workers, research scientists, volunteers, managers, receptionists and nurses.

Funding: Core funding for ALSPAC is provided by the UK Medical Research Council and Wellcome (Grant ref: 102215/2/13/2) and the University of Bristol. Genotyping of the ALSPAC maternal samples was funded by the Wellcome Trust (WT088806) and the offspring samples were genotyped by Sample Logistics and Genotyping Facilities at the Wellcome Trust Sanger Institute and LabCorp (Laboratory Corporation of America) using support from 23andMe. This work has also been supported by the US National Institute of Health (R01 DK10324), the European Research Council under the European Union's Seventh Framework Programme (FP7/2007-2013) / ERC grant agreement no 669545, the British Heart Foundation (AA/18/7/34219) and the NIHR Biomedical Centre at the University Hospitals Bristol NHS Foundation Trust and the University of Bristol. A comprehensive list of ALSPAC grant funding is available on the ALSPAC website (http://www.bristol.ac.uk/alspac/external/documents/grant-acknowledgements.pdf). DAL and MCB work in a unit that receives funding from the UK Medical Research Council (MC_UU_00011/6). JPK is funded by a University of Queensland Development Fellowship (UQFEL1718945), and his research was carried out at the Translational Research Institute, Woolloongabba, QLD 4102, Australia. The Translational Research Institute is supported by a grant from the Australian Government. MCB is supported by a UK Medical Research Council Skills Development Fellowship (MR/P014054/1) and DAL is an NIHR Senior Investigator (NF-0616-10102).

deCODE
We thank the participants in the Icelandic deCODE study, the staff at deCODE genetics core facilities and the staff at the Research Service Center for their important contribution to this work.

DNBC
We are very grateful to the women taking part in the DNBC. The DNBC was established with the support of a major grant from the Danish National Research Foundation. Additional support for the DNBC has been obtained from the Danish Pharmacists' Fund, the Egmont Foundation, the March of Dimes Birth Defects Foundation, the Augustinus Foundation and the Health Fund of the Danish Health Insurance Societies. The DNBC biobank is a part of the Danish National Biobank resource, which is supported by the Novo Nordisk Foundation. B.F. was supported by the Oak Foundation.

FINNPEC
The FINNPEC study was supported by Jane and Aatos Erkko Foundation, Paivikki and Sakari Sohlberg Foundation, Academy of Finland, Research Funds of the University of Helsinki, Government special state subsidy for health sciences at the Hospital District of Helsinki and Uusimaa, Finska Läkaresällskapet, Liv och Hälsa Foundation, Novo Nordisk Foundation, Finnish Foundation for Pediatric Research, Emil Aaltonen Foundation, Sigrid Juselius Foundation, and Finnish Foundation for Laboratory Medicine.

GOPEC
The GOPEC collection was funded by the British Heart Foundation Programme Grant RG/99006.

HUNT

The Nord-Trøndelag Health Study (The HUNT Study) is a collaboration between HUNT Research Centre (Faculty of Medicine and Health Sciences, Norwegian University of Science and Technology NTNU), Nord-Trøndelag County Council, Central Norway Health Authority, and the Norwegian Institute of Public Health.

MoBa
The Norwegian Mother, Father and Child Cohort Study is supported by the Norwegian Ministry of Health and Care Services and the Ministry of Education and Research. We are grateful to all the participating families in Norway who take part in this on-going cohort study and to Ms Anita Haugan for her work on the project. Genotyping of the MoBa Preeclampsia case-control population was supported by NIH/NICHD (R01HD058008).

SSI
The SSI study of severe, early-onset preeclampsia in Danish women and their offspring was funded by a grant from the Danish Council for Independent Research (1331-00240B).

Wellcome Trust Sanger Institute
We thank scientific and administrative staff at the Wellcome Trust Sanger Institute for their support for this work, especially those in Human Genetics, Genotyping, DNA Sequencing, and Sample Management. We also gratefully acknowledge Wellcome Trust support for this work under Wellcome Trust grant 098051.

This publication is the work of the authors and V.S. and R.M will serve as guarantors for the contents of this paper. The views expressed in this paper are those of the authors and not necessarily, any funding body or anyone acknowledged.

## Supplementary References

[1] Holly F Ainsworth, Jennifer Unwin, Deborah L Jamison, and Heather J Cordell. Investigation of maternal effects, maternal-fetal interactions and parent-of-origin effects (imprinting), using mothers and their offspring. Genetic epidemiology, 35(1):19–45, 2011.

[2] Richard Howey, Chrysovalanto Mamasoula, Ana Töpf, Ron Nudel, Judith A Goodship, Bernard D Keavney, and Heather J Cordell. Increased power for detection of parent-of-origin effects via the use of haplotype estimation. The American Journal of Human Genetics, 97(3):419–434, 2015.
