# Supplementary material for: Genetic predisposition to hypertension is associated with preeclampsia in European and Central Asian women
Source: Nat Commun. 2020 Nov 25;11:5976. doi: 10.1038/s41467-020-19733-6 (PMC7688949; doi:10.1038/s41467-020-19733-6)
Supplement: Supplementary file 3 — Description of Additional Supplementary Files [file 41467_2020_19733_MOESM3_ESM.pdf]

Description of Additional Supplementary Files

File Name: Supplementary Data 1.

Description: Summary of functional annotations for FLT1 preeclampsia risk locus.

File Name: Supplementary Data 2.

Description: Follow up of variants with $P < 1E-6$ in the maternal meta-analysis.

File Name: Supplementary Data 3.

Description: Blood pressure variants that associate with preeclampsia with $P < 0.05$.

File Name: Supplementary Data 4.

Description: EMIM analysis of maternal and fetal effect.
