# Supplementary material for: Genetic predisposition to hypertension is associated with preeclampsia in European and Central Asian women
Source: Nat Commun. 2020 Nov 25;11:5976. doi: 10.1038/s41467-020-19733-6 (PMC7688949; doi:10.1038/s41467-020-19733-6)
Supplement: Supplementary file 8 — Reporting Summary [file 41467_2020_19733_MOESM8_ESM.pdf]

nature research

Corresponding author(s): Valgerdur Steinthorsdottir; Ralph McGinnis

Last updated by author(s): Sep 30, 2020

Reporting Summary

Nature Research wishes to improve the reproducibility of the work that we publish. This form provides structure for consistency and transparency in reporting. For further information on Nature Research policies, see Authors & Referees and the Editorial Policy Checklist .

Statistics

For all statistical analyses, confirm that the following items are present in the figure legend, table legend, main text, or Methods section.

n/a	Confirmed	
☐	☒	The exact sample size (*n*) for each experimental group/condition, given as a discrete number and unit of measurement
☒	☐	A statement on whether measurements were taken from distinct samples or whether the same sample was measured repeatedly
☐	☒	The statistical test(s) used AND whether they are one- or two-sided<br>*Only common tests should be described solely by name; describe more complex techniques in the Methods section.*
☐	☒	A description of all covariates tested
☐	☒	A description of any assumptions or corrections, such as tests of normality and adjustment for multiple comparisons
☐	☒	A full description of the statistical parameters including central tendency (e.g. means) or other basic estimates (e.g. regression coefficient) AND variation (e.g. standard deviation) or associated estimates of uncertainty (e.g. confidence intervals)
☐	☒	For null hypothesis testing, the test statistic (e.g. *F*, *t*, *r*) with confidence intervals, effect sizes, degrees of freedom and *P* value noted<br>*Give P values as exact values whenever suitable.*
☒	☐	For Bayesian analysis, information on the choice of priors and Markov chain Monte Carlo settings
☒	☐	For hierarchical and complex designs, identification of the appropriate level for tests and full reporting of outcomes
☐	☒	Estimates of effect sizes (e.g. Cohen's *d*, Pearson's *r*), indicating how they were calculated

Our web collection on statistics for biologists contains articles on many of the points above.

Software and code

Policy information about availability of computer code

Data collection	No specific computer code was used for data collection
Data analysis	We used PLINK (version 1.90b), EIGENSOFT (6.0.1), Admixture (version 1.23), SHAPEIT (version 2), IMPUTE2 (v2.3.2), SNPTEST (v2.5), METAL (version released 24 February 2009), GCTA (v1.91.1 beta), LDSC (v1.0.0), LocusZoom (v1.4) and R (v3.6.0) to analyse the data and produce plots for this manuscript.

For manuscripts utilizing custom algorithms or software that are central to the research but not yet described in published literature, software must be made available to editors/reviewers. We strongly encourage code deposition in a community repository (e.g. GitHub). See the Nature Research guidelines for submitting code & software for further information.

Data

Policy information about availability of data

All manuscripts must include a data availability statement. This statement should provide the following information, where applicable:

- Accession codes, unique identifiers, or web links for publicly available datasets
- A list of figures that have associated raw data
- A description of any restrictions on data availability

Meta-analysed GWAS data used in this study as well as individual-level GWAS data from the Uzbek and Kazakh studies and whole-genome sequencing data on Uzbek and Kazakh subjects have been deposited in the European Genome-phenome Archive (https://www.ebi.ac.uk/ega) under the accession numbers listed below.
Whole Genome Sequencing:
EGAD00001005467 [https://www.ebi.ac.uk/ega/datasets/EGAD00001005467],
EGAD00001005466 [https://www.ebi.ac.uk/ega/datasets/EGAD00001005466]
Kazakhstan GWAS Genotypes:
EGAD00010001945 [https://www.ebi.ac.uk/ega/datasets/EGAD00010001945]

EGAD00010001949 [https://www.ebi.ac.uk/ega/datasets/EGAD00010001949],
EGAD00010001947 [https://www.ebi.ac.uk/ega/datasets/EGAD00010001947]
Uzbekistan GWAS Genotypes:
EGAD00010001917 [https://www.ebi.ac.uk/ega/datasets/EGAD00010001917],
EGAD00010001918 [https://www.ebi.ac.uk/ega/datasets/EGAD00010001918],
EGAD00010001919 [https://www.ebi.ac.uk/ega/datasets/EGAD00010001919]
GWAS Meta-Analyses:
EGAD00010001983 [https://www.ebi.ac.uk/ega/datasets/EGAD00010001983],
EGAD00010001984 [https://www.ebi.ac.uk/ega/datasets/EGAD00010001984],
EGAD00010001985 [https://www.ebi.ac.uk/ega/datasets/EGAD00010001985],
EGAD00010001986 [https://www.ebi.ac.uk/ega/datasets/EGAD00010001986],
EGAD00010001987 [https://www.ebi.ac.uk/ega/datasets/EGAD00010001987],
EGAD00010001988 [https://www.ebi.ac.uk/ega/datasets/EGAD00010001988].

For pre-computed LD scores for European populations, see https://data.broadinstitute.org/alkesgroup/LDSCORE/eur_w_ld_chr.tar.bz2; for ENCODE project see www.encodeproject.org.

Field-specific reporting

Please select the one below that is the best fit for your research. If you are not sure, read the appropriate sections before making your selection.

☒ Life sciences ☐ Behavioural & social sciences ☐ Ecological, evolutionary & environmental sciences

For a reference copy of the document with all sections, see nature.com/documents/nr-reporting-summary-flat.pdf

Life sciences study design

All studies must disclose on these points even when the disclosure is negative.

Sample size	This is a meta-analysis of several GWAS studies on preeclampsia and all available samples, that passed QC, were includes. Based on the number of cases and controls included, the power of the study to detect association for a given effect size and variant frequency was estimated and is included in supplementary material.
Data exclusions	Genetic variants and samples were excluded from the study based on predefined quality checks described in the methods section. For some of the included cohorts individuals were excluded from the control groups if they had hypertension.
Replication	No replication was performed. This is a meta-analysis of multiple GWAS studies with additional data added for selected variants.
Randomization	Not relevant for this study, as this is a case-control GWAS study
Blinding	Not relevant for this study, as this is a case-control GWAS study

Reporting for specific materials, systems and methods

We require information from authors about some types of materials, experimental systems and methods used in many studies. Here, indicate whether each material, system or method listed is relevant to your study. If you are not sure if a list item applies to your research, read the appropriate section before selecting a response.

Materials & experimental systems

n/a	Involved in the study	
☒	☐	Antibodies
☒	☐	Eukaryotic cell lines
☒	☐	Palaeontology
☒	☐	Animals and other organisms
☐	☒	Human research participants
☒	☐	Clinical data

Methods

n/a	Involved in the study	
☒	☐	ChIP-seq
☒	☐	Flow cytometry
☒	☐	MRI-based neuroimaging

Human research participants

Policy information about studies involving human research participants

Population characteristics	See Supplementary Table 13 for population characteristics and phenotype distributions of participating studies
Recruitment	Participants from Central Asia were recruited for this study. Women of Uzbek and Kazakh ancestry, were recruited between 2012 and 2015 from maternity units in Uzbekistan and Kazakhstan. Women with singleton pregnancies affected by preeclampsia were recruited at the time of diagnosis; healthy pregnant controls were recruited from the same maternity centres. Other participants were recruited originally as a part of numerous studies, described in Supplementary Table 1 and Supplementary Table 13, each of which had different selection criteria as described in the Methods section.
Ethics oversight	The protocols for individual studies included in this work were approved by: The Derbyshire Research Ethics Committee; The Icelandic National Bioethics Committee; the ALSPAC Ethics and Law Committee and Local Research Ethics Committees; The Regional Committee for Medical Research, South East Norway; The Scientific Ethics Committee of the Danish Capital City Region; The Helsinki University Hospital ethical committee; The Ministry of Health, Republic of Kazakhstan, Central Ethics Committee; The National Ethics Committee, Ministry of Health, Republic of Uzbekistan; The Hospital District of Helsinki and Uusimaa Co-ordinating Ethics Committee; The Regional Committee for Medical and Health Research Ethics, Central Norway and The North West Research Ethics Committee (UK).

Note that full information on the approval of the study protocol must also be provided in the manuscript.
